# A roadmap to understanding diversity and function of coral reef-associated fungi

**DOI:** 10.1093/femsre/fuac028

**Published:** 2022-06-23

**Authors:** Anna Roik, Miriam Reverter, Claudia Pogoreutz

**Affiliations:** Helmholtz Institute for Functional Marine Biodiversity, University of Oldenburg, Ammerländer Heerstraße 231, 26129 Oldenburg, Germany; Institute for Chemistry and Biology of the Marine Environment, Carl von Ossietzky University of Oldenburg, Wilhelmshaven, 26046, Germany; Alfred Wegener Institute, Helmholtz Centre for Polar and Marine Research (AWI), Am Handelshafen 12, 27570 Bremerhaven, Germany; Institute for Chemistry and Biology of the Marine Environment, Carl von Ossietzky University of Oldenburg, Wilhelmshaven, 26046, Germany; School of Biological and Marine Sciences, University of Plymouth, Plymouth PL4 8AA, United Kingdom; Laboratory for Biological Geochemistry, School of Architecture, Civil and Environmental Engineering, École Polytechnique Fédérale de Lausanne (EPFL), 1015 Lausanne, Switzerland

**Keywords:** marine fungi, interkingdom interactions, nutrient cycling, ecosystem functioning, chemical mediation, probiotics

## Abstract

Tropical coral reefs are hotspots of marine productivity, owing to the association of reef-building corals with endosymbiotic algae and metabolically diverse bacterial communities. However, the functional importance of fungi, well-known for their contribution to shaping terrestrial ecosystems and global nutrient cycles, remains underexplored on coral reefs. We here conceptualize how fungal functional traits may have facilitated the spread, diversification, and ecological adaptation of marine fungi on coral reefs. We propose that functions of reef-associated fungi may be diverse and go beyond their hitherto described roles of pathogens and bioeroders, including but not limited to reef-scale biogeochemical cycles and the structuring of coral-associated and environmental microbiomes *via* chemical mediation. Recent technological and conceptual advances will allow the elucidation of the physiological, ecological, and chemical contributions of understudied marine fungi to coral holobiont and reef ecosystem functioning and health and may help provide an outlook for reef management actions.

## Teaser

Conceptual perspective on known and proposed functions of understudied marine fungi on coral reefs and in reef-building corals, their main ecosystem engineers.

## Introduction

### The coral reef: a microbially driven ecosystem

Tropical coral reefs are highly diverse and productive ecosystems shaped by their main ecosystem engineers, reef-building corals. Corals are “holobionts,” multicellular animal hosts associated with a diverse suite of prokaryotes and microeukaryotes (Rohwer et al. 2002). The best studied host–microbe interaction in these holobionts is the coral–dinoflagellate symbiosis, a reciprocal nutrient-exchange relationship (Muscatine and Porter 1977). This symbiosis has formed the very foundation of the ecological success of coral reefs over hundreds of millions of years, and its breakdown can rapidly lead to host morbidity and death (Rädecker et al. 2021). While our understanding of the coral–dinoflagellate symbiosis builds upon decades of extensive research (Davy et al. 2012), other coral and reef-associated microbiota are presumed to be of importance for holobiont and ecosystem functioning as well, but their roles remain poorly understood.

Recently, functional studies on coral-associated prokaryotes and their role in host–microbe interactions have gained traction focusing on nitrogen cycling pathways (Rädecker et al. 2015), sulfur cycling, specifically in the context of dimethylsulfoniopropionate (DMSP; Glossary) transformations (Raina et al. 2010a), antioxidant (Dungan et al. 2021), and antibiotic activities (Ritchie 2006). Increasingly, genomic studies elucidating the functional diversity of coral bacteria suggest some prokaryotes may be drivers of coral holobiont functioning, resilience, and ecological adaptation (Vega Thurber et al. 2009, Neave et al. 2017, Pogoreutz et al. 2022), and marine probiotic applications are currently being explored for bioremediation and reef restoration purposes (Rosado et al. 2018, Doering et al. 2021).

Corals and other reef holobionts are also home to members of Archaea and other microeukaryotes, including fungi (Wegley et al. 2007, Ainsworth et al. 2017). The enigmatic Kingdom of Fungi is considered an ecological driving force that shapes terrestrial ecosystems (including some of the harshest ecosystems on Planet Earth; Coleine et al. 2022) and global biogeochemical cycles by interconnecting different levels of biological and ecological organization (Bahram and Netherway 2022). Yet, on coral reefs, studies on the taxonomic and functional diversity of fungi have been rare and far between.

### Aims of this review

Fungi in the marine realm, and on coral reefs in particular are understudied compared to terrestrial and freshwater ecosystems (Bärlocher and Boddy 2016). We here interpret the available knowledge on coral reef-associated fungi in the light of fungal functional traits and ecological niches in different ecosystems to propose a conceptual perspective of fungal interactions on coral reefs. We cover a spectrum of putative functions and ecological interactions based on fungal cellular, physiological, metabolic, and molecular traits to illustrate their manifold ecological potential. Based on this, we conceptualize how these functional traits may have facilitated the spread, diversification, and ecological adaptation of fungi in coral reef environments. We propose that reef-associated fungi are functionally and metabolically diverse and might contribute to coral reef biogeochemical cycles potentially impacting multiple levels of biological organization, ranging from the cellular to holobiont and, ultimately, the reef scale *via* benthic–pelagic coupling (Glossary). We further discuss the potential spectrum of interactions of fungi with other organisms on and around reefs, ranging from mutualism and commensalism to parasitism. Based on these comparisons, we hypothesize that fungi may play a pivotal role in the health and ecological functioning of coral reefs, and in reef-building coral holobionts in particular. Finally, we conclude our work with future research directions that we hope will stimulate the advancement of research of fungi on coral reefs.

### Abundance and microhabitats of marine fungi and their diversity on coral reefs

#### Fungal abundance and microhabitats

Environmental substrate availability is a major driver of abundance and biomass of marine fungi (Clipson et al. 2006). Not surprisingly, fungal cell numbers and biomasses are much lower in the open ocean compared to sediments and terrestrial ecosystems, their occurrence likely being restricted to association with particles (Wurzbacher et al. 2010). Yeasts in the pelagic zone of oligotrophic lakes or coastal environments exhibit low cell densities typically below one colony-forming unit (CFU) ml^–1^ up to 47 CFU ml^–1^ in hypertrophic systems (Woollett and Hedrick 1970, Libkind et al. 2003). In highly productive coastal upwelling systems, fungi can exhibit similar biomasses as heterotrophic prokaryotes, thereby significantly contributing to the living microbial carbon (C) and nitrogen (N; Gutiérrez et al. 2011). While no information on fungal abundances on coral reefs are currently available, the typically oligotrophic conditions would suggest low environmental abundances of pelagic fungi, which may increase under eutrophication or dissolved organic carbon enrichment, as observed for copiotrophic bacteria (known as “microbialization’ of coral reefs; Haas et al. 2016; Glossary).

In oligotrophic aquatic systems, expected to support only low to moderate fungal biomass, fungal contributions to ecosystem nutrient cycling may be of lesser significance than that of prokaryotes. Fungal metabolism becomes potentially relevant in very specific scenarios (summarized in Wurzbacher et al. 2010), such as in stagnant microhabitats, biofilms, on surfaces, and environments characterized by steep spatial gradients (Sampaio et al. 2007); on algae (Kagami et al. 2007a); in aggregates and extracellular polysaccharides (Masters 1971); in the presence of highly recalcitrant (Glossary) nutrient sources that require specific enzymes to be metabolized (Reisert and Fuller 1962, Fischer et al. 2006); as part of symbiotic associations (Whisler et al. 1975, Gimmler 2001, Ibelings et al. 2004) and predator–prey relationships (Barron 1996).

**Figure 1.**
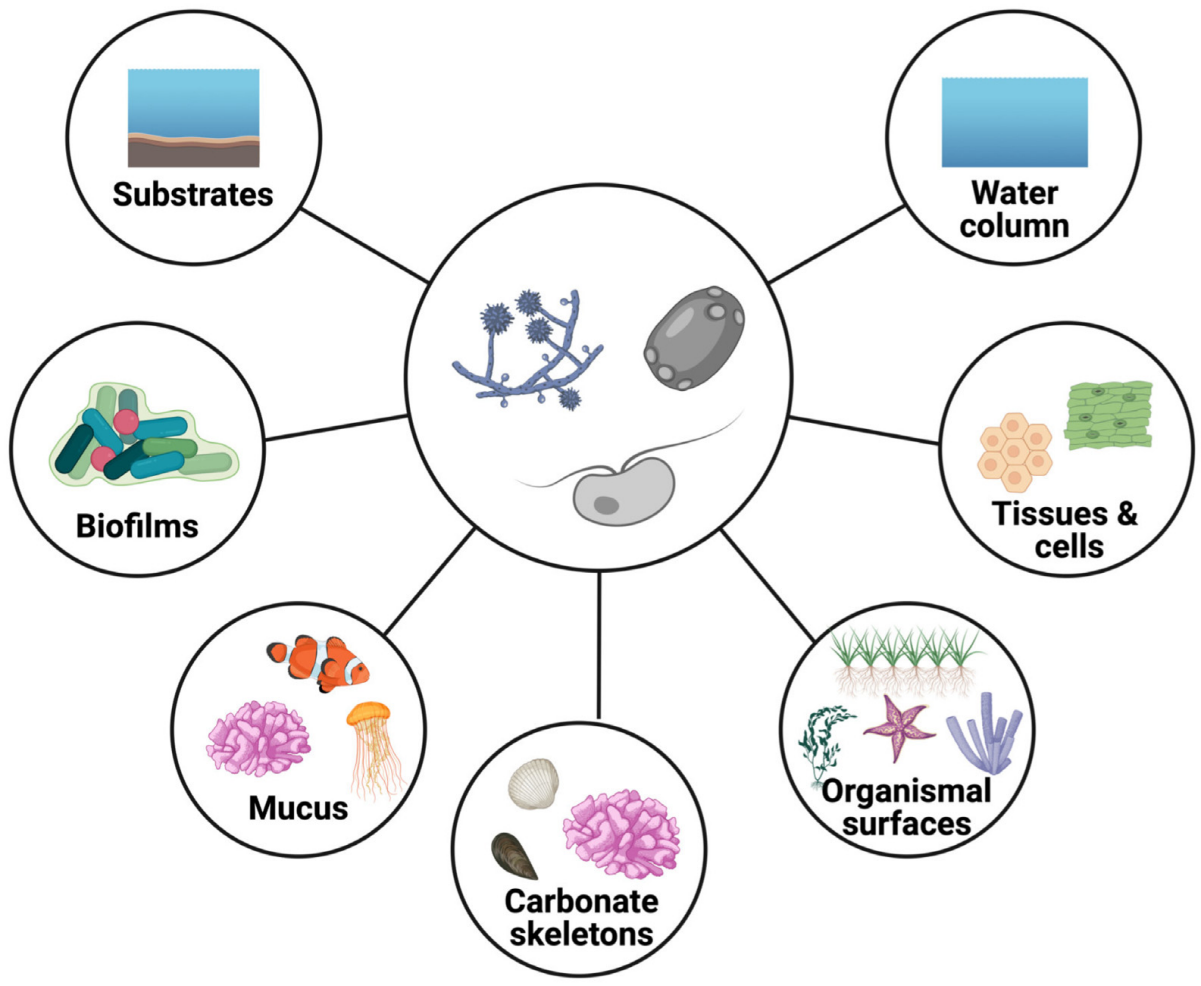
Potential microhabitats of fungi on coral reefs. Marine fungi likely inhabit diverse microhabitats on coral reefs, such as substrates (rock, rubble, and interstitial spaces in sediments) and biofilms that have formed on reef substrates (Sampaio et al. 2007), but also the water column, where fungi may be predominantly associated with particles or planktonic organisms (Wurzbacher et al. 2010). Mucosal spaces of reef invertebrates, in particular Cnidaria, but also vertebrates such as fish could potentially attract fungi (Reisert and Fuller 1962, Fischer et al. 2006, Nelson et al. 2013). Furthermore, any animal epithelial or macrophyte surface (e.g. seagrasses and macroalgae) may harbor fungal communities (Kagami et al. 2007a, Ettinger and Eisen 2019). Aside from surface colonization, endolithic fungi have been reported from calcium carbonate skeletons of corals and the reef framework (Risk and Muller 1983, Priess et al. 2000). Finally, fungi may be associated with the tissues or cells of macro-organisms (Nadal et al. 2008, Trofa et al. 2008). The center represents different stages and/or forms of fungal cells. The outer circles represent potential microhabitats of fungi on the coral reef.

On coral reefs, multiple if not all these scenarios may apply at varying spatial scales ranging from the cellular over the holobiont to, potentially, the ecosystem scale (Fig. 1). While pelagic fungi might not be very abundant on coral reefs, reefs harbor diverse and abundant benthic substrata suitable for fungal colonization such as the reef framework and rubble along with reef sediments. Coral skeletons underneath the living tissues constitute stagnant microhabitats characterized by (micro)surfaces, porous matrices, and steep gradients of light, oxygen, pH, and nutrients (Risk and Muller 1983, Schlichter et al. 1997, Venn et al. 2011, Wangpraseurt et al. 2012). Further, coral reefs harbor a diversity of potential uni- and multicellular hosts fungi could associate with (Fig. 1). Coral tissues and skeletons are populated by microalgae such as the Symbiodiniaceae (Dinoflagellata; Davy et al. 2012) and *Ostreobium* (Chlorophyta), respectively, as well as by prokaryotes and fungi (Bentis et al. 2000, Rohwer et al. 2002), so that diverse mutualistic, antagonistic, and/or synergistic microbe–microbe interactions could arise. Finally, corals constantly secrete mucus containing high levels of recalcitrant dissolved organic C and extracellular polysaccharides (Nelson et al. 2013), resulting in aggregate formations in the water column that contribute to reef energy transfer and nutrient cycles (Wild et al. 2004). Taken together, we hypothesize that coral reefs potentially harbor a diversity of fungi that might exhibit numerous functions in the pelagic and benthic communities, which is discussed in detail in the following sections.

### Diversity

**Figure 2.**
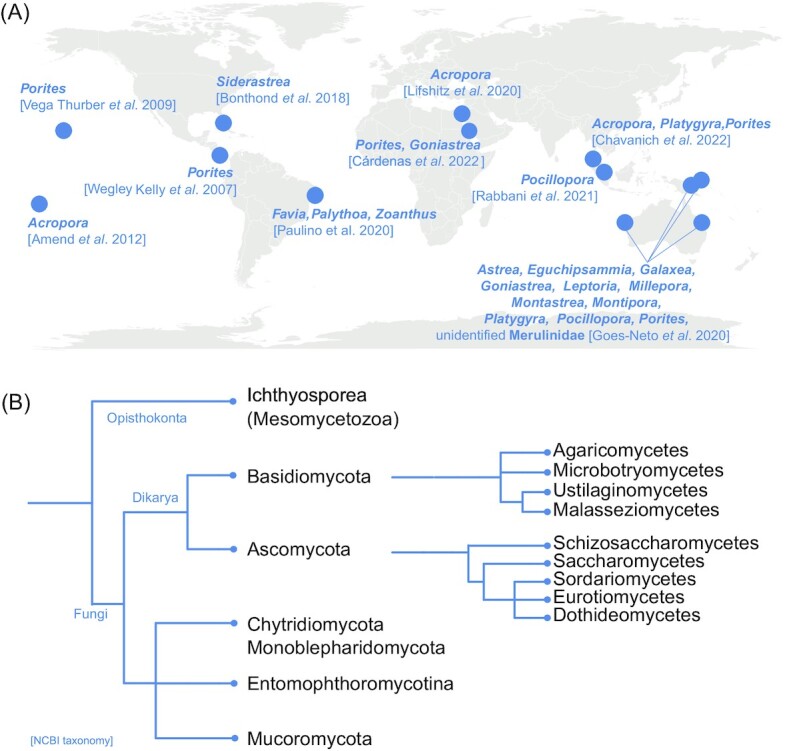
Overview of fungal diversity studies in corals. **(A)** Geographic distribution of sampling sites and investigated coral genera (created using *maps* package in *R*). **(B)** Pruned phylogenetic trees displaying consistently reported fungal phyla (and classes for Ascomycetes and Basidiomycetes) across studies (NCBI taxonomy; generated using *phyloT v2*, Letunic 2015).

Little is known about the diversity, ecology, and evolution of animal-associated (Bahram and Netherway 2022) and marine fungi, including coral- and reef-associated fungi (Golubic et al. 2005, Amend et al. 2019, Gladfelter et al. 2019). The few fungal diversity studies available for coral holobionts (collated in Table S1, Supporting Information) represent an appreciable geographic spread of sampling locations (Fig. 2). At the same time, they reflect the well-known constraints of phylogenetic markers and/or genomic databases available for fungi (Frau et al. 2019, Rabbani et al. 2021), and hence do not permit definitive statements on the specificity of the coral-associated fungal community at lower taxonomic ranks (Table S2, Supporting Information). However, these sampling efforts so far provide a valuable first glimpse into coral-associated fungal communities. Of these, most studies assessed entire corals without separation into surface mucus layer, tissues, and skeleton (e.g. Chavanich et al. 2022). A subset of studies characterized fungal communities in coral mucus and tissues separately from the coral skeleton (Bonthond et al. 2018, Rabbani et al. 2021), while others focused entirely on the skeleton, and/or limestone reef substrates (Kohlmeyer and Volkmann-Kohlmeyer 1989, Kohlmeyer et al. 2000; Góes-Neto et al. 2020, Cárdenas et al. 2022; Table S2, Supporting Information). Consequently, we cannot currently extract more specific information regarding potential compartmentalization of fungal communities within the coral holobiont. However, we highlight notable taxa consistently reported in association with coral holobionts between these studies.

High proportions of Ascomycota in the culturable fraction of coral fungal isolates (Lifshitz et al. 2020, Paulino et al. 2020) and in fungal sequencing data are apparent, sometimes in excess of 85% relative abundance (Wegley et al. 2007, Góes-Neto et al. 2020, Cárdenas et al. 2022), although dominances of Basidiomycota or Chytridiomycota (Glossary) sequences were reported for endolithic communities (Glossary) of some corals (Góes-Neto et al. 2020; Fig. 2). The most commonly reported ascomycetes in corals are Sordariomycetes, notably *Lindra* (Lulworthiales), *Hyalorhinocladiella* (Ophiostomatales), and *Physalospora* spp. (Xylariales; Vega Thurber et al. 2009, Amend et al. 2012, Bonthond et al. 2018, Góes-Neto et al. 2020). Further, Hypocreales, as well as Dothideomycetes, Eurotiomycetes, and Saccharomycetes (Wegley et al. 2007, Lifshitz et al. 2020, Paulino et al. 2020, Rabbani et al. 2021, Cárdenas et al. 2022) are consistently reported across coral species and ocean basins (Table S1, Supporting Information). Most notably, sequences affiliated to *Hortaea* spp. including *H*. *werneckii* in the order Dothideomycetes were consistently reported (Amend et al. 2012, Bonthond et al. 2018, Rabbani et al. 2021, Cárdenas et al. 2022), the latter being an emerging model organism for osmotolerance studies (please refer to *“Fungal Traits”*). Within the Eurotiomycetes, notable representatives are *Aspergillus* spp. or *Penicillium* spp. (Wegley et al. 2007, Lifshitz et al. 2020, Paulino et al. 2020, Rabbani et al. 2021, Chavanich et al. 2022). Members of the Basidiomycota are commonly reported from corals at low relative abundances, and include Ustilaginomycetes, Agaricomycetes, Microbotryomycetes, and Malasseziomycetes (Wegley et al. 2007, Bonthond et al. 2018, Lifshitz et al. 2020, Paulino et al. 2020, Rabbani et al. 2021). Yet, while “truly” marine fungi are considered those able to grow and/or sporulate in marine environments, to form symbiotic relationships with marine organisms, to adapt and evolve at the genetic level, and/or be metabolically active in marine environments (Pang et al. 2016), disentangling true marine indwellers from fungi stemming from terrestrial input or laboratory contamination remains a challenge (Amend 2014).

Few studies have investigated the community dynamics of coral-associated fungal communities in response to environmental change. While it appears that coral-associated fungal communities might be host-specific (Cárdenas et al. 2022, Chavanich et al. 2022), they are extremely diverse and heterogeneous, which may mask further subtle community differences shaped by the environment (Amend et al. 2012, Bonthond et al. 2018, Rabbani et al. 2021). As such, no geographical patterns of coral-associated fungal communities have been apparent so far (Rabbani et al. 2021), but a greater phylogenetic diversity and heterogeneity of fungi in acroporid corals was reported for reefs in warmer compared to cooler waters (Amend et al. 2012) as well as in corals exhibiting tissue lesions (Lifshitz et al. 2020). Further, increased abundances of sequences affiliated to Saccharomycetes and Malasseziomycetes (Chavanich et al. 2022) and reduced abundances of Sordariomycetes and Agaricomycetes were reported in bleached or heat-stressed) corals, respectively (Cárdenas et al. 2022). Finally, coral-associated fungal metagenomic sequences were shown to increase and/or shift toward zoosporic members under environmental stress suggesting fungal proliferation (Wegley et al. 2007, Vega Thurber et al. 2009, Góes-Neto et al. 2020). Importantly, while information on the diversity of associated fungal communities can be considered scarce, even less information is available regarding fungal functional traits and their interactions on coral reefs and with(in) (coral) holobionts (Ainsworth et al. 2017, Gladfelter et al. 2019). In this light, two major fungal groups have received attention in the past: first, putative pathogens and opportunists such as *Aspergillus sydowii*, a fungus linked to sea fan aspergillosis resulting in large scale mortality (Smith et al. 1996); second, endolithic, i.e. skeleton-associated fungi of reef-building corals (Kendrick et al. 1982, Golubic et al. 2005, Fig. 3). The state of knowledge on these two most widely studied groups of reef-associated fungi is briefly summarized below (refer to *fungal parasites, pathogens*, and*opportunists*).

**Figure 3.**
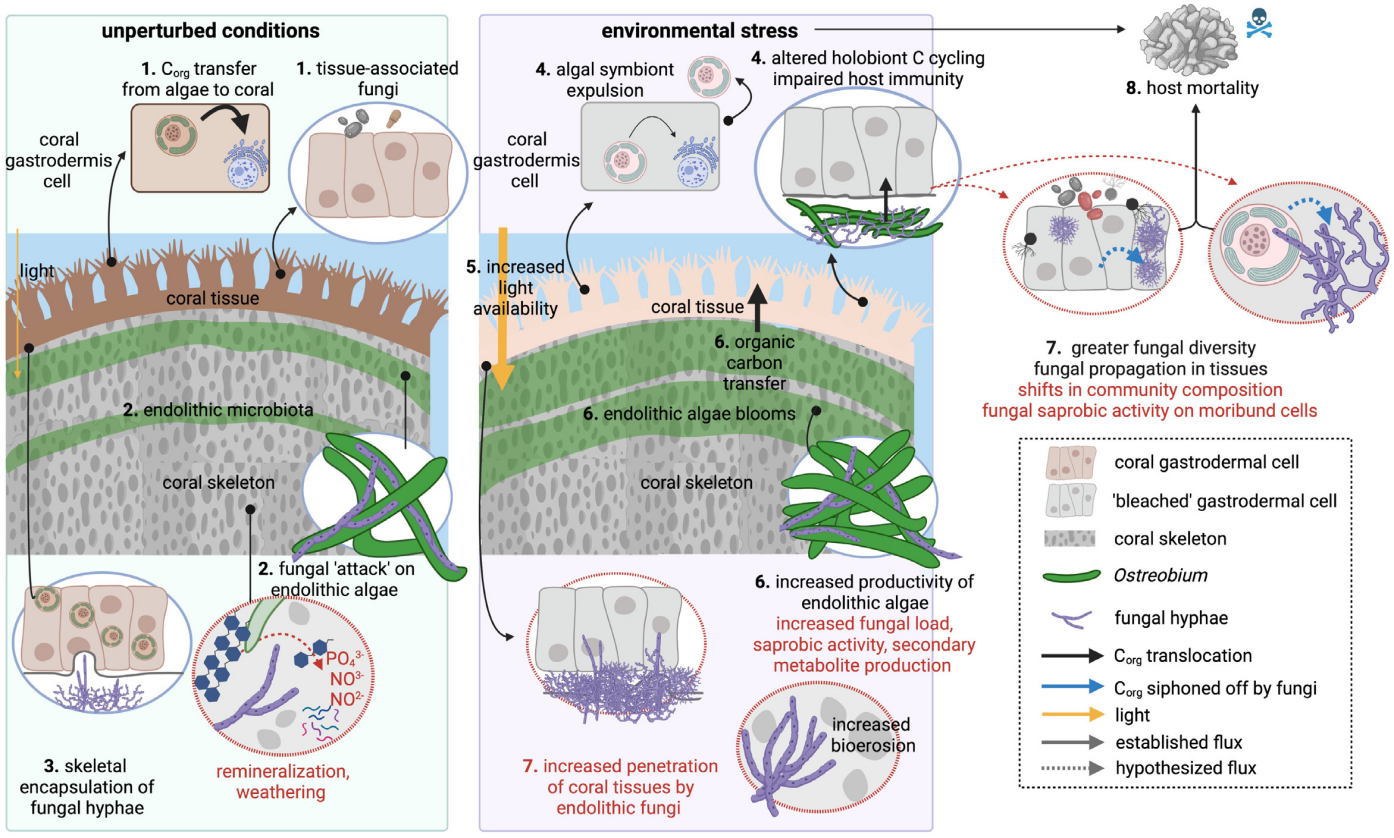
Established and proposed holobiont–fungal interactions in reef-building corals. Corals are complex holobionts comprised of distinct functional compartments: the tissues (gastrodermis and ectodermis), algal endosymbionts (Symbiodiniaceae; localized in host gastrodermal cells), and the skeleton, which harbors a diverse microbiome including the filamentous alga *Ostreobium*. Under unperturbed conditions (left panel), coral tissues receive large amounts of organic carbon (C_org_) from Symbiodiniaceae (1). Endolithic fungi coreside with the filamentous algae *Ostreobium* inside the coral skeleton where they form visible banding patterns (2). Endolithic fungi may “attack” *Ostreobium* cells but are unable to penetrate healthy host tissues, which form skeletal protuberances around fungal hyphae (Bentis et al. 2000) (3). Endolithic fungi may perform organic matter remineralization and mineral weathering, resulting in high inorganic nutrient concentrations in skeletal pore water (Risk and Muller 1983) (3). Under environmental stress (right panel), coral tissues experience a disruption of algal endosymbiont C_org_ translocation and subsequently expel Symbiodiniaceae, resulting in tissue paling (“coral bleaching”), altered holobiont nutrient cycling, and impaired host immunity (Rädecker et al. 2021) (4). Coral bleaching results in transparent host tissues and allows more light to penetrate into the skeleton (5), resulting in blooms of endolithic phototrophs and C_org_ translocation from the endoliths to the coral tissues (Fine and Loya 2002) (6). Environmental stress may increase diversity and proliferation of coral-associated fungi (Vega Thurber et al. 2009; Amend et al. 2012) (6), and increased skeletal erosion (Yarden et al. 2007). Thermal stress and weakening of host immune responses may result in opportunistic growth and saprobic activity of coral-associated fungi, which may be accompanied by fungal lifestyle switching (7). In a severely immunocompromised host, fungal infection of tissues and remaining algal endosymbionts may exacerbate holobiont health (Strake et al. 1988), leading to host mortality (Alker et al. 2001) (8). Arrows without dash represent established fluxes, dashed arrows represent hypothesized fluxes. Blue fluxes refer to hypothesized C_org_ fluxes to fungi. Black text refers to established activity and interactions. Red text refers to proposed interactions. Blue dashed arrows refer to the proposed diverting of C_org_ to fungi.

### Fungal traits and potential relevance on coral reefs

The ocean environment is starkly different from terrestrial ecosystems. In order to better understand and resolve the putative roles of fungi in marine environments and reef-associated holobionts, it is hence critical to consider the cellular, physiological, metabolic, and genomic traits fungi are equipped with (Table S3, Supporting Information). These traits have allowed fungi to conquer diverse niches and ecosystems, including some of the harshest known environments (Coleine et al. 2022). Fungi can go airborne *via* spore dispersal, utilize airborne compounds for growth, or stimulate plant growth *via* volatile compounds (Vespermann et al. 2007, Naznin et al. 2013). In aquatic ecosystems, fungi occupy a diversity of ecological niches, and can exhibit a diversity and biomass comparable to that of prokaryotes (Gutiérrez et al. 2011). Fungi are found in rather contrasting environments from sewage treatment plants to ultraoligotrophic conditions, such as in water distillation apparatuses (summarized in Wurzbacher et al. 2010). Ultimately, the ability for lichenization (not further discussed in this review), i.e. fungal partners engaging in photosymbiosis with algae, render fungi the ultimate pioneers of barren, harsh environments (Spribille et al. 2016). This versatility of fungi regarding dispersal and habitat colonization can be attributed to a range of functional traits and adaptations as described below, some of which may be fundamental to help them thrive in marine environments including coral reefs, and to potentially engage in complex symbioses.

### Fungal cell wall properties, cellular integrity, and osmotolerance

Fungal cells have peculiar characteristics, which may be relevant for survival in the ocean. Their cell walls consist of multiple layers of polysaccharides (Szaniszlo and Mitchell 1971, Durán and Nombela 2004), which render the cell highly stable and water absorbent. Interestingly, differences in cell wall compositions between ecologically restricted terrestrial and marine species exhibit quantitative, but not qualitative differences in carbohydrate, amino sugar, amino, and fatty acid composition (Szaniszlo and Mitchell 1971, Ravishankar et al. 2006, Plemenitaš et al. 2014, Danilova et al. 2020). Further, marine fungi were shown to produce enzymes involved in fatty acid modifications to maintain cell wall fluidity and integrity (Turk et al. 2004, Kogej et al. 2007, Gostincar et al. 2009). Another feature of cell walls in aquatic fungi is the incorporation of melanin (Cordero and Casadevall 2017), which increases cell strength, rigidity, and tolerance to hydrostatic pressure, high UV radiation, and osmotolerance (Casadevall et al. 2017, Cordero and Casadevall 2017), properties relevant for adaptation to marine environments.

Ocean salinity (0.6 M NaCl) is considered a potential barrier to fungal growth (El Baidouri et al. 2021). Hence, osmolytes likely play a central role in fungal adaptation to marine environments (Danilova et al. 2020, Gonsalves and Nazareth 2020). Across marine environments, osmotolerance in fungi is variable, with some species exhibiting strong local adaptation to (hyper)saline environments (Kohlmeyer and Kohlmeyer 2013, Buchalo et al. 2019). Particularly high halotolerance has been reported for yeasts such as *H. werneckii* (Hohmann et al. 2007), an emerging fungal model organism able to grow in environments up to 5 M NaCl (Plemenitaš et al. 2014). Adaptations of *H. werneckii* to high osmolarity beyond the discussed cell wall properties include ion accumulation (Kogej et al. 2005) and modifications in the high-osmolarity-glycerol (HOG) signaling pathway (Turk and Plemenitas 2002), which controls the regulation of the osmolyte glycerol (Muzzey et al. 2009). The HOG signal transduction pathway is rapidly activated following cell shrinking under hyperosmotic shock, initiating inorganic ion export (Proft and Struhl 2004), cell cycle arrest (Escoté et al. 2004), diminished translation (Bilsland-Marchesan et al. 2000), closure of glycerol export channels (Tamás et al. 2003), and activation of glycolysis to counteract cell shrinking (Dihazi et al. 2004). Hog1 is deactivated once the cell commences reswelling due to glycerol accumulation (Hohmann et al. 2007). In addition to glycerol, halophilic and halotolerant fungi produce diverse pools of osmolytes such as saccharides, polyols, melanin, mycosporine-like amino acids, and unidentified UV-absorbing compounds (Kogej et al. 2006, Ravishankar et al. 2006, Danilova et al. 2020). Often, multiple osmolytes are produced in response to hyperosmotic shock, and the composition of osmolyte pools differs with fungal identity, growth phase (Kogej et al. 2007), and environmental pH (Gonsalves and Nazareth 2020).

Finally, the genomes of aquatic fungi and yeasts encode for high numbers of major enzymes involved in cellular oxidative stress responses such as superoxide dismutases, catalases, and peroxiredoxins (Gostinčar and Gunde-Cimerman 2018). These enzymes are central to mounting antioxidant responses in high Na+, low K+ environments (Gostinčar and Gunde-Cimerman 2018), which may in part explain the “Phoma pattern” (Ritchie 1959), the correlation of osmotolerance with thermotolerance (Prista et al. 2005). Some marine fungi genomes are further characterized by a high G + C content (e.g. *Emericellopsis atlantica*; Hagestad et al. 2021), a feature previously linked to complex environmental adaptation and horizontal gene transfer (HGT; Mann and Chen 2010) and halotolerance in prokaryotes (Jacob 2012).

### Unicellularity and dimorphic switching facilitating the aquatic and host-associated lifestyle

Plasticity of morphological and lifestyle traits has allowed fungi to colonize a variety of environmental niches through different strategies (Větrovský et al. 2019). While multicellular, filamentous hyphal networks are common in terrestrial habitats, fungi that colonize sugar-rich plant-associated microhabitats such as fruit and nectar, aquatic environments, or uni- and multicellular eukaryotic hosts including intestinal environments tend to rely on unicellular, yeast-like lifestyles, and/or dormant spores (Andrews et al. 1994, Nagy et al. 2017). Even more successful are fungi with the ability to reversibly switch between the hyphal multicellular and unicellular form (Boyce and Andrianopoulos 2015). Dimorphic switching (Glossary) has been observed in many terrestrial ascomycetes that are known pathogens in insect or mammalian hosts including humans, but are also able to survive in free-living forms. Dimorphic switching in pathogens is triggered by environmental cues, primarily temperature (Pasricha et al. 2017, Francisco et al. 2019). The switch to a unicellular lifestyle typically involves the compositional remodeling of the hyphal cell wall (characterized by mannoproteins, glucans, and chitin) to evade detection by the host's immune system (Klis et al. 2009, Nagy et al. 2017). By these means and *via* nitric oxide reductases and other antioxidants, many dimorphic fungi can modulate the host immune response and proliferate intracellularly within host phagocytes (Holbrook et al. 2011, Nagy et al. 2017, Chandrasekar et al. 2022).

While many dimorphic fungi are infectious in their unicellular stage, others, such as human-associated commensal *Candida* spp., can invade and harm immunosuppressed hosts through switching from unicellular to their hyphal stage, which causes damage by penetrating tissues through filamentous growth (Trofa et al. 2008). Similarly, most plant pathogens become pathogenic during their hyphal stage, which enables the fungus to invade the plant tissues (Nadal et al. 2008). In corals, endolithic fungi seemingly attempt invasion of coral tissues from the calcareous skeleton underneath (Bentis et al. 2000), and hyphae-like cells co-occur with morbid, disease-like host phenotypes (Strake et al. 1988, Work et al. 2008). These fungi potentially spread and infect corals as free-swimming yeast-like forms or may switch to hyphal growth to opportunistically invade immunosuppressed, stressed corals. Finally, dimorphic switching might not only facilitate opportunistic or parasitic interactions, but potentially the establishment of mutualistic symbioses, as observed during lichenization of *Umbilicaria muhlenbergi* (Wang et al. 2020). Overall, the ability of fungi to switch between unicellular and multicellular forms facilitates potentially numerous strategies to survive diverse environments and to engage in interkingdom interactions. While such strategies remain to be discovered in the marine realm, the capacity for dimorphic switching to modulate immune responses for host invasion could explain the prevalence and ubiquity of fungi across marine hosts and habitats. As such, fungal characteristics involved in dimorphic switching will be an interesting trait to investigate to reveal yet unknown mechanisms of coral–fungal interactions.

### Motility, chemotaxis, and attachment

Some unicellular aquatic fungi exhibit a degree of motility. Members of the ancestral lineage Chytridiomycota, or chytrids have adapted to “the life aquatic” *via* active swimming, specifically targeting new substrates and hosts by producing high numbers of motile asexual zoospores (van Hannen et al. 1999). Motility in chytrids is mediated by chemotactic behavior (Glossary: chemotaxis) toward specific amino acids and carbohydrates (Muehlstein et al. 1988, Scholz et al. 2017). Specific cell structures, including the chytrid *rumposome*, a complex of interconnecting tubules connecting the cell surface with the flagellar apparatus, are implicated in the zoospore response to environmental signals (Powell 1983). In contrast to motile zoospores, most fungi including yeasts are nonmotile and typically require substrates to grow on. Attachment strategies to such substrates are hence important and facilitated by the production of mucilaginous sheaths, expression of surface proteins, called flocculins (Ogawa et al. 2019), or spore walls (Jones 2006), as reported from some red and black yeasts (Andrews et al. 1994). Extracellular polysaccharides are associated with enhanced growth under oligotrophic conditions and may bind both ionic and nonionic nutrients (Kimura et al. 1998). Lectins, a group of carbohydrate binding proteins are primarily present in the cell wall of aquatic yeasts and implicated in aggregation and adhesion to substrates (Singh et al. 2011), specifically attaching to polysaccharides on the cell walls of hosts, or to detritus (summarized in El Baidouri et al. 2021). A diversity of adhesion strategies allows for the direct connection between filamentous fungi with yeasts resulting in the formation of structures, so called biocapsules, in the liquid environment (Ogawa et al. 2019), which could help facilitate attachment in the ocean.

### Nutrient acquisition strategies

Diverse and highly effective nutrient acquisition strategies are one of the major hallmarks of fungal metabolism. These include exceptional enzymatic capabilities. Major groups of enzymes produced by fungi are relevant for the decomposition and degradation of recalcitrant organic matter, thereby playing an important role in ecosystems *via* the regeneration of C and N sources. Marine and freshwater chytrids are widely assumed to employ a range of extracellular enzymes as part of their diverse secretome, including carbohydrate-active enzymes (CAZymes; Glossary; Lange et al. 2019). A range of fungal enzymes target humic acids or polymers such as lignin, (hemi)celluloses, tunicin (Kohlmeyer and Kohlmeyer 2013, Castaño et al. 2021), or chitin (Tang et al. 2006). The latter occurs in high abundances not only in terrestrial but also aquatic ecosystems, e.g. as part of arthropod exoskeletons and fungal cell walls (Reisert and Fuller 1962).

The marine realm is home to many unique substrates either not found in terrestrial environments, or subject to modifications such as sulfation, i.e. the addition of sulfate groups, the removal of which is necessary for substrate utilization (Janusz et al. 2017, Schultz-Johansen et al. 2018, Barbosa et al. 2019, Kappelmann et al. 2019). This includes algal-derived complex polysaccharides, including but not limited to laminarin, fucoidan, porphyrin, and chitin. The broad substrate range observed in some marine fungi (Thomas et al. 2022) is likely related to the diverse battery of CAZymes they harbor, such as glycoside hydrolases, which render fungi capable of degrading otherwise recalcitrant polysaccharides. Generalists such as *Emericellopsis atlantica* tend to harbor a higher diversity of CAZymes than specialists permitting the degradation of a greater range of substrates (Zhao et al. 2014b, Hagestad et al. 2021). Importantly, a high diversity of CAZymes and broad substrate range may convey high adaptive capacity to different hosts or substrates, are likely implicated in the diversification of nutritional modes (Janusz et al. 2017) and suggest marine fungi may act as vectors of organic matter transfer within marine food webs (Thomas et al. 2022). Ultimately, a broad substrate range may be beneficial for adaptation to oligotrophic marine environments, such as coral reefs. In oligotrophic environments, most marine fungi may seek out and adapt to specific niches where nutrients and/or organic matter are “concentrated,” such as the environment of uni- and multicellular hosts of the coral reef benthos. Sponges for instance are filter feeders that efficiently remove particulate and dissolved organic matter from tons of cubic meters of seawater per hour, and are known hosts to marine fungi (Anteneh et al. 2019). Pelagic systems, however, are likely inhabited by parasitic and saprobic fungi such as chytrids, which infect phytoplankton hosts and draw from their pool of photosynthetic organic carbon (Klawonn et al. 2021) or degrade particulate organic matter (Roberts et al. 2020). Of note, the expression of fungal chitinases, peptidases, and relatives of β-N-acetylglucosaminidases has been reported in reef-building corals (Amend et al. 2012), suggesting similar lifestyles as in the water column.

### Fungi as secondary metabolite producers

Fungi produce a plethora of structurally unique bioactive compounds that have evolved as key molecules in fungal chemical communication, defense, and competition, facilitating interactions with hosts and other microorganisms (Kusari et al. 2012, Bahram et al. 2018, Keller 2019). Fungal metabolites exhibit numerous antibacterial, antifungal, antiviral, and anticancer bioactivities, which have long attracted interest in fungi as a source of new drugs (Keller 2019). In fact, the first antibiotic molecule in history, penicillin, was discovered nearly a century ago from the culturable fungus *Penicillium notatum* (Wong 2003). Another prominent example includes the potent anticancer compound paclitaxel (taxol), which is widely used in the treatment of different types of cancer, and which was initially isolated in 1993 from an endophytic fungus (*Taxomyces adrenae*) associated with Pacific yew trees (*Taxus brevifolia*; Stierle et al. 1993). An increasing interest in the untapped chemical diversity of marine fungi has arisen during the last years (Agrawal et al. 2018, Liu et al. 2019). Many marine fungi associated with algae and marine invertebrates such as sponges and corals have been shown to produce a broad diversity of metabolites with varied bioactivities (El-Gendy et al. 2018, Bovio et al. 2019, Kamat et al. 2020, Peng et al. 2021). However, despite the increasing number of studies investigating marine fungal metabolites and bioactivities, their biological and ecological roles remain largely unknown.

Secondary metabolite synthesis often relies on primary metabolite pools (i.e. initial building blocks), which feed into specialized biosynthetic pathways involving large multimodular enzymes such as polyketide synthases PKSs, nonribosomal peptide synthetase NRPSs, prenyltransferases, and terpene cyclases (Brakhage and Schroeckh 2011, Keller 2019). Genes encoding these enzymes are arranged in Biosynthetic Gene Clusters (BGCs; Glossary; Brakhage and Schroeckh 2011). Given the high energy cost of secondary metabolite production, fungi have evolved effective strategies to control the expression of BGCs (Shostak et al. 2020). Many BGCs in monocultured fungi are often silent, and their expression is highly dependent upon environmental and biotic stimuli (Brakhage and Schroeckh 2011, Netzker et al. 2015). For example, the phytopathogenic fungus *Sclerotinia sclerotiorum* activates different BGCs when infecting different hosts (Allan et al. 2019) and the fungal BGC encoding the production of the antibacterial compound bikaverin is only activated when exposed to metabolites from the bacterial competitor *Ralstonia solanacearum* (Spraker et al. 2018).

The remarkable flexibility of fungal metabolism has hindered the understanding of their biological roles and modes of action, especially in marine fungi. However, the development of new tools allowing the study of metabolites *in situ* (e.g. MALDI-tof; Glossary) and the use of genome mining approaches to identify BGCs has tremendously increased our knowledge in recent years (Boya et al. 2017, Medema et al. 2021). Although this blooming field has so far focused on unsilencing BGCs for drug discovery purposes (Brakhage and Schroeckh 2011), much can be learned and applied for the ecological study of fungi and will without doubt provide new opportunities to better understand the roles that fungal secondary metabolites play in coral reefs and holobionts.

### Rapid adaptive evolution of fungal genomes

Fungal genomes vary greatly regarding their organization, composition, and ploidy levels. While typically small and dynamic, genome sizes range from around 2 Mb (similar to those of many bacteria) in the unicellular parasitic Microsporidia to around 2 Gb in Pucciniales (rust fungi), in the same order of magnitude as the human genome (Stajich 2017). Fungal genomes (those of pathogens in particular) have an extraordinary capacity for rapid evolution reflected in distinct genome compositions and compartmentalization, extensive sequence divergence, and distinct chromosome organization (Möller and Stukenbrock 2017, Stajich 2017, summarized in Feurtey and Stukenbrock 2018), along with an abundance of transposable elements (Hess et al. 2014, Miyauchi et al. 2020, Gluck-Thaler et al. 2022), evidence for diversifying selection linked to environmental adaptation, niche specialization, and host–microbe interactions (Sperschneider et al. 2015). Further, there is increasing evidence for interspecific gene exchange through hybridization or frequent HGT and viral transfer (HVT; Bian et al. 2020, Wang et al. 2021b, Gluck-Thaler et al. 2022). These mechanisms are poorly explored in fungi, may occur between highly distinct species of fungi (Soanes and Richards 2014) and nonfungal organisms including hosts, and have been predominantly studied in pathogenic terrestrial lineages (Friesen et al. 2006, Menardo et al. 2016).

Gene exchange *via* hybridization occurs sexually or asexually (Roper et al. 2011, Stukenbrock 2016), typically during secondary contact of fungal propagules, and can give rise to novel adaptive traits and adaptive capacity with new ecological niches and hosts (Soanes and Richards 2014, Feurtey and Stukenbrock 2018). This includes the rapid evolution of host specificities and virulence phenotypes (Stukenbrock et al. 2012, Menardo et al. 2016, Silva et al. 2018). Similarly, HGT/HVT between fungi, other eukaryotes, bacteria, and viruses may not only drive rapid adaptive fungal evolution, but also mediate switches from pathogenic to nonpathogenic lifestyles (Zhou et al. 2021). Rates of prokaryotic HGT differ between fungal lineages, with proportions of prokaryotic HGT events ranging from none in the Saccharomycetales up to 65% of investigated cases in the Pezizomycotina (Marcet-Houben and Gabaldón 2010). HGT with nonfungal eukaryotes include interactions with insect and plant hosts (Zhao et al. 2014a), but are unexplored in the marine realm. Importantly, as genes involved in the same metabolic pathways are often physically clustered in the genome (Wisecaver and Rokas 2015), the acquisition of (partial) gene clusters *via* HGT/HVT can extend the physiological repertoire of a recipient organism by providing complete, novel metabolic pathways (Feurtey and Stukenbrock 2018).

While more research is required, the here described capacity for rapid adaptive evolution may not be limited to pathogenic lineages and may help facilitate the adaptation and radiation of fungi to new niches in the marine realm, such as pelagic or interstitial environments including sediments, coral skeletons, and different hosts on coral reefs. Coordinated efforts to increase the availability of genomic sources of coral reef-associated fungi will help elucidate the genetic underpinnings of marine fungal adaptation.

### Genome functional gene content in marine fungi

The survival of microorganisms in oligotrophic marine environments requires the evolution of diverse transporters and catalysts capable of functioning under an alkaline pH and ionic stress (Moran et al. 2004, Bonugli-Santos et al. 2015). While little information is available for marine fungi, a similar observation was made in the model yeast *Dendryphiella hansenii*. Compared to terrestrial yeasts, its genome is particularly enriched with genes for C and N transport, but also for multidrug resistance (Lépingle et al. 2000). *Dendryphiella hansenii* has numerous examples of gene duplications in conjunction with reductions in the proportion of noncoding DNA and the shortening of overall gene lengths. This results in similar genome sizes, but different genomic coding densities in marine and nonmarine yeasts (coding densities of 79.2% and 70.3% in genome sizes of around 13 Mb in *D*. *hansenii* and *S*. *cerevisiae*, respectively). The observed gene duplications may reflect the requirements of a more demanding environment, such as a marine habitat, which selects for the retention of duplicated genes even when resulting changes in encoded protein activities are very slight (Dujon et al. 2004).

### Transcriptional features of fungi

Changes to transcriptional activity (Glossary: transcription) during certain stages in the fungal life cycle may produce phenotypic variation in response to fluctuating or changing environments, which may be conducive to survival and acclimation. Conidiation, the formation of conidiophores from vegetative hyphae, is one such critical stage. Wang et al. (2021a) reported that conidia in *Aspergillus nidulans, A*. *fumigatus*, and *Talaromyces marneffei* exhibited transcriptional activity while still in the conidiophore, and synthesized mRNA until their release and dormancy was established. Conidia exhibit environment-specific transcriptional responses to temperature shock, osmotic shock, or zinc deficiency, which affects conidial content (mRNAs, proteins, and secondary metabolites). This in turn affects the fitness and capabilities of fungal cells after germination, stress and antifungal resistance, mycotoxin and secondary metabolite production, and virulence (Wang et al. 2021a). Thereby, the conidia synthesize and store transcripts according to prevalent environmental conditions. Some freshwater fungal lineages were proposed to have evolved from terrestrial fungi in part due to their sticky drifting, branched conidiospores which may easily attach to submerged substrates (Grossart et al. 2019). While this remains yet to be confirmed, it may be plausible that not only drifting dormant spores, but entire conidiophores of terrestrial or freshwater fungi may be relocated into the ocean *via* run-off. Maintaining transcriptional activity, displaced, drifting conidia still developing may be able to attain acclimation *via* “front-loading” of conidial content before being released from the conidiophore. Such physiological peculiarity could help explain the activity, acclimatization, and in the long run adaptation and diversification of fungal lineages in marine environments, including coral reefs.

Finally, little is known about host–fungi symbioses and their underlying molecular mechanisms of symbiosis establishment and maintenance on coral reefs. Major changes in host–symbiont gene (co-) expression reflecting genetic reprogramming and modulation of molecular crosstalk may facilitate novel associations, as reported for arbuscular mycorrhizal fungi symbioses (Glossary; Mateus et al. 2019). While no mycorrhiza-like fungal symbioses on coral reefs are currently known, molecular tools such as dual RNA-seq technology may help elucidate the nature of marine host–fungi relationships and identify putative key genes associated with symbiosis establishment, as previously employed for other poorly understood coral–microbe associations (Mohamed et al. 2018).

### Fungal ecology in the context of coral reefs

Fungi are recognized for their role as major conduits mediating the transfer of energy and nutrients through terrestrial food webs (Azam 1998, Moore et al. 2004). While fungi-mediated organic matter transformation and nutrient cycling processes in the ocean are less understood (Amend et al. 2019), we know different ecological guilds (Glossary) of fungi occur in the ocean, such as saprotrophs (Cunliffe et al. 2017, Hagestad et al. 2021), parasites (Laundon et al. 2021), and putative pathogens (Smith et al. 1996, Yarden et al. 2007). At the land–ocean interface, endophytic and mycorrhizal associations with plants are known from salt marshes (Newell 1996, Clipson et al. 2006). On coral reefs however, no comparable examples of mutualistic host–microbe or microbe–microbe interactions of fungi have been reported yet. Rather, studies are skewed toward opportunistic, pathogenic, or parasitic interactions due to their environmental impact (Bentis et al. 2000, Alker et al. 2001, Sweet et al. 2013). Fungal interactions on coral reefs are hypothesized to include interspecies (fungal–fungal; Bärlocher and Kendrick 1974) or interkingdom interactions (fungal–prokaryote and fungal–eukaryote; Golubic et al. 2005). The extent of these interactions likely varies with functional traits of the interacting partners as well as abiotic factors (Cheeke et al. 2017, Francisco et al. 2019). In the light of functional traits of fungi from different ecosystems including marine fungi, we argue that fungi may be relevant for coral reef ecosystem functioning at different levels of biological organization and spatial scales. In the following sections, we discuss potential scenarios in which fungi could exert beneficial functions in the context of mediating biogeochemical cycles, and potential mutualistic organismal interactions on coral reefs. This is followed by examples of known and hypothesized pathogenic, opportunistic, and parasitic interactions.

### Fungal contributions to biogeochemical cycling in the ocean: a metabolic black box

Marine fungi likely contribute to the remineralization of recalcitrant organic matter and processes significant for the cycling of C, N, phosphorus (P), and sulfur (S) in marine systems (Gutiérrez et al. 2011, 2020). Marine fungi harbor an extensive battery of suitable exoenzymes, which may result in high substrate affinity and broad substrate range (Newell 1996, Zhao et al. 2014b, Hagestad et al. 2021, Thomas et al. 2022; see Section *Fungal functional traits—Nutrient acquisition strategies*, where the fungal secretome is introduced). Thereby, marine fungi may help mobilize organic C in the ocean *via* the remineralization of recalcitrant high molecular weight detritus, thereby diverting energy to higher trophic levels through saprobic (Gutiérrez et al. 2011, 2020, Thomas et al. 2022) and parasitic routes (Klawonn et al. 2021). Beyond C, however, our knowledge on marine fungal biogeochemical cycling remains obscure.

**Figure 4.**
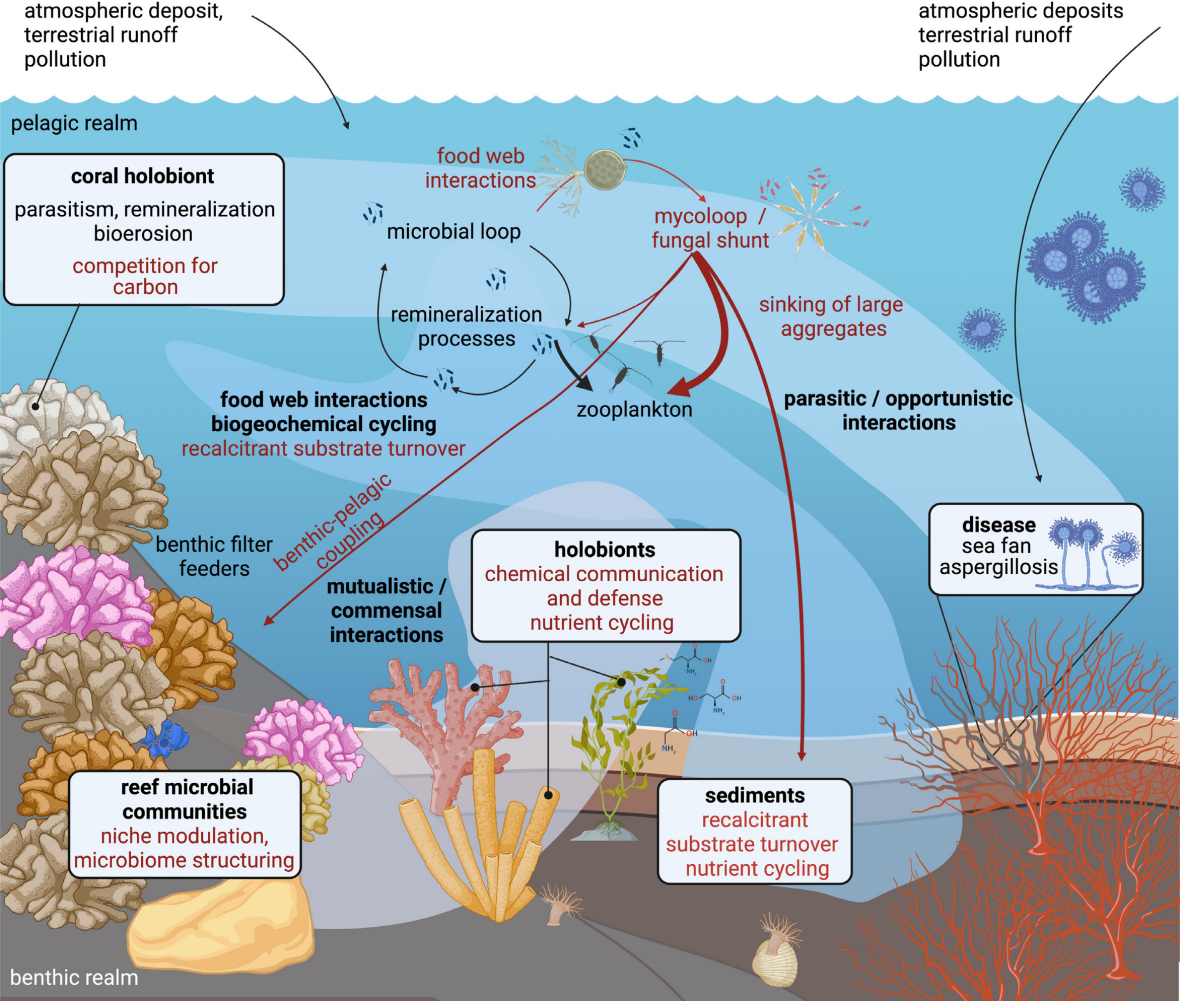
Synthesis of known (black arrows) and proposed (red arrows) interactions and functions of fungi associated with the coral holobiont and the coral reef ecosystem. The most obvious and best-studied fungal interactions on coral reefs include putative parasitism, pathogenesis, and bioerosion. Based on fungal functions in terrestrial and other aquatic ecosystems, we propose that reef-associated fungi may further play roles in the structuring of holobiont- and reef-associated microbial communities and biogeochemical cycling.

Of particular interest is N, a major limiting element in the oligotrophic ocean, including coral reefs (Cardini et al. 2015, Rädecker et al. 2015, Pogoreutz et al. 2017). N is essential for the growth and activity of marine fungi (Clipson et al. 2006), which may satisfy much of their N requirements from the degradation of photosynthates (Dring and Dring 1992) and recalcitrant polymeric compounds including chitin in the molts and carapaces of marine crustaceans (Kirchner 1995, Tang et al. 2006). Endophytic and mycorrhizal fungi likely account for nearly all N present in the decaying standing plant biomass on salt marshes (Newell 1996, Clipson et al. 2006), while fungal rather than bacterial denitrification is a major driver of N_2_O production in redox-dynamic coastal sediments in the German Wadden Sea (Wankel et al. 2017). Although intertidal Wadden Sea and subtidal coral reef sediments will starkly differ in their (a)biotic properties, reef sediments are a place of significant microbial turnover of organic matter such as partially recalcitrant coral mucus aggregates. Mineralization of coral mucus fuels benthic and pelagic productivity on coral reefs *via* the release of limiting inorganic nutrients such as N and P (Wild et al. 2004, 2005). Fungi may contribute to such coral mucus remineralization processes *via* the turnover of other recalcitrant organic matter in reef sediments (Fig. 4). In corals, fungal N metabolism was suggested to help prevent N loss from the holobiont (Rädecker et al. 2015). Indeed, fungal genes associated with N metabolism and transport are well represented in coral-associated metagenomes and fungal mRNA transcripts. These genes are related to the metabolism of nucleic acids, amines, and cellular nitrogen compounds, as well as enzymes involved in urea, glutamate, glutamine, and ammonification pathways (Wegley Kelly et al. 2007; Amend et al. 2012). It was further proposed that fungal N metabolism might partially account for the high levels of inorganic N concentrations in the interstitial pore water in coral skeletons, where septate fungi can be abundant (Le Campion-alsumard et al. 1995).

Fungal P and S cycling properties in the ocean remain largely unknown. The macronutrient P commonly occurs at very low concentrations in the open ocean and in oligotrophic coastal ecosystems, such as tropical coral reefs. P concentrations limit oceanic bacterial productivity (Van Wambeke et al. 2002), help maintain marine photosymbioses (Wiedenmann et al. 2012, Rädecker et al. 2015) and are a primary driver of pelagic marine thraustochytrid distribution and biomass across space and time (Bongiorni and Dini 2002). Fungi could potentially contribute to P cycling *via* remineralization processes in reef sediments and coral skeletons (Risk and Muller 1983, Wild et al. 2004) or by primary mineral weathering as observed in terrestrial ectomycorrhizal fungi (Landeweert et al. 2001). Both could in part explain comparatively high phosphate levels in the pore water of coral skeletons (Risk and Muller 1983, Fig. 3). Importantly, fungal nutrient release could help alleviate nutrient limitation at small spatial scales (mm to cm) for other organisms within the coral skeleton (*Ostreobium*; prokaryotes) or potentially even the tissues (coral host, Symbiodiniaceae, prokaryotes; Fig. 3).

Different (in)organic S compounds (including sulfides and methanethiol) are readily metabolized by different marine fungal isolates, suggesting a tentative contribution to coral reef S cycling (Wainwright 1989, Faison et al. 1991, Phae and Shoda 1991, Bacic and Yoch 1998). Of particular interest may be DMSP transformations, as reflected in the degradation of DSMP from algae and salt-marsh grass *Spartina alterniflora* by *Fusarium lateritium* (Bacic and Yoch 1998) and the presence and activity of a DMSP lyase implicated in DMSP catabolism (Glossary) in the coral pathogen *A. sydowii* (Kirkwood et al. 2010). Considering the potential ecological relevance of DMSP as osmolyte and antioxidant in corals (Raina et al. 2009), fungal DMSP transformations could be of importance in the holobiont.

In conclusion, fungi may contribute to coral reef biogeochemical cycling, albeit at likely varying spatial scales and levels of biological organization (Figs 3 and 4). Experimental approaches aiming to elucidate fungal biogeochemical cycling on coral reefs from the cellular to the ecosystem level may draw from a diversity of novel analytical tools enabling the study of cell-to-cell level interactions to broad ecological questions (cf. *Challenges, open questions and future directions*).

### Fungal decomposition activity as driver of microbiome structure and function

Fungi and bacteria share numerous microhabitats where they form dynamic, coevolving assemblages (Deveau et al. 2018). Such close spatial coexistence in the environment or within complex holobionts gives rise to a spectrum of interactions ranging from antagonistic to synergistic (Glossary; Bengtsson 1992, Mille-Lindblom et al. 2006). Antagonistic interactions may be based on interference competition involving allelochemicals (i.e. chemicals produced by living organisms that affect physiological processes in other organisms), such as in early stages of host infection and substrate colonization (Mille-Lindblom et al. 2006). Synergistic interactions may include the provisioning of “public goods:” fungi may release resources *via* the generation of more accessible, intermediate decomposition products of recalcitrant organic matter, which bacteria (or other organisms) cannot access on their own (Tang et al. 2006, Schneider et al. 2012, Roberts et al. 2020). Metaproteomic analysis of microbial leaf litter decomposing communities showed that the majority of proteins affiliated to extracellular hydrolytic enzymes were related to fungi, and none to bacterial hydrolases (Schneider et al. 2012). Strong positive correlation of bacterial abundances with fungal extracellular enzymes suggested bacterial “cheating behavior” (Velicer 2003) by exploiting low molecular weight carbohydrates from fungal decomposition (Boer et al. 2005, Schneider et al. 2012). Such fungal–bacteria interactions can affect host and ecosystem health and functioning by structuring microbiome community composition (Boer et al. 2005, McFrederick et al. 2014, Bahram et al. 2018). Indeed, saprobic chytrids decomposing chitin particles were found to alter the associated bacterial community structure and diversity (Roberts et al. 2020), which may be related to fungal processing of recalcitrant organic matter into more readily accessible C (Tang et al. 2006, Cunliffe et al. 2017, Roberts et al. 2020, Thomas et al. 2022). Coral reef-associated pelagic and benthic fungi may exhibit similar roles as decomposers, thereby contributing to bacterial community structuring, colonization and succession in complex holobionts and the environment, ultimately shaping ecosystem biogeochemical cycling (as suggested for pelagic chytrids: Roberts et al. 2020, Klawonn et al. 2021, Fig. 4). Manipulative studies leveraging metaproteomic and metabolomic approaches combined with coculturing and next generation sequencing applications (Glossary) may help elucidate this intriguing prospect of fungal interactions in pelagic and benthic coral reef environments.

### Ecological interactions through chemical mediation

Chemical communication between organisms is one of the most primitive and widespread languages in nature, and a major driver of biological complexity. Bacteria and fungi are widely recognized for their roles in shaping ecosystems through the production of semiochemicals (i.e. chemical substances released by an organism that affect the behavior of other organisms; Davis et al. 2013, Ditengou et al. 2015). These metabolites govern many intra- and interspecific interactions, and while some provide collective benefits (i.e. public goods, such as in biofilm formation or quorum sensing), others shape communities through antagonism (Hogan 2006, Schoenian et al. 2011). Despite the importance of microbial chemical mediation in diverse, complex ecosystems such as coral reefs, this remains a highly underexplored research area. In this section, we provide different examples of fungal chemical mediation that may be of relevance to coral holobiont and coral reef ecosystem functioning, with the aim to identify research gaps and new potential research avenues.

#### Quorum sensing

Quorum sensing (QS), a concerted, density-dependent cell-to-cell signaling process, is one of the most widely studied chemical communication strategies in fungi (Hogan 2006, Barriuso et al. 2018, Tian et al. 2021). Unicellular fungi produce QS molecules that accumulate in the surrounding environment during the growth of the population. When cell concentrations exceed a threshold, the QS molecules trigger coordinated population processes such as virulence/pathogenesis, morphological differentiation, sporulation, secondary metabolite production, and enzyme secretion (Barriuso et al. 2018). While several QS molecules have been characterized (e.g. terpenes, lactones, alcohols, peptides, and oxylipins), the structure, specificity, and mode of action of many fungal QS molecules remain puzzling (Affeldt et al. 2012, Barriuso et al. 2018). For example, farnesol (12-carbon sesquiterpene alcohol) has been considered a broad-spectrum QS molecule capable of eliciting dimorphic switching and hyphal growth in phylogenetically diverse fungi including *Candida albicans, Ophiostoma piceae*, and *Penicillium decumbens* (Hornby et al. 2001, Guo et al. 2011, de Salas et al. 2015). However, in other *Ophiostoma* species, *O. ulmi and O. floccosum*, dimorphic switching is mediated by 2-methyl-1-butanol and cyclic sesquiterpenes (Berrocal et al. 2012, 2014). Butyrolactones have also been identified in different fungi (i.e. *Penicillium sclerotiorum* and *Aspergillus terreus*) as QS molecules capable of regulating the production of antimicrobial metabolites (Raina et al.2010b, 2012). In other *Aspergillus* species, oxylipins (i.e. lipids created from fatty acid oxidation; Glossary) regulate morphological differentiation and mycotoxin production (Tsitsigiannis et al. 2005, Horowitz Brown et al. 2008, Affeldt et al. 2012). In addition to population cooperation, QS molecules also play important roles in interspecific interactions and cross-kingdom signaling (Sztajer et al. 2014, Dixon and Hall 2015). For example, farnesol acts as an antimicrobial agent in both bacteria and fungi (Derengowski et al. 2009), and can disrupt bacterial QS communication (Cugini et al. 2007). In coral reefs, the mechanisms underpinning fungal QS and the potential role of these molecules in other ecosystem interactions and processes remain poorly understood. Interestingly, coral fungal endophyte extracts were found to inhibit bacterial QS (Martín-Rodríguez et al. 2014), suggesting that fungi are likely to play important ecological roles such as influencing microbiome assembly, structuring, and antifouling protection in coral holobionts (Fig. 4).

#### Oxylipins

Oxylipins (Glossary) are ubiquitous in a wide range of organisms, play major roles in biological processes, e.g. regulating inflammation and cellular homeostasis in metazoans. Oxylipins are major mediators of cross-kingdom talk and host–fungal interactions that encompass predator–prey, mutualistic, and pathogenic and biological processes (Holighaus and Rohlfs 2019, Niu and Keller 2019). For example, the fungal volatile oxylipin 1-octen-3-ol is found in the feces of *Aspergillus*-infested beetle larvae, which in turn is used by parasitoid wasps as a cue to detect unfavorable environments (i.e. mold-infested beetles; Steiner et al. 2007). This repellant response was found to be innate in the wasps, suggesting that host-associated fungi may be important in parasitoid host-finding strategies (Steiner et al. 2007). Fungal oxylipins can also alter oxylipin production in plant and mammalian hosts in order to modulate or attenuate responses of the host immune system, and hence facilitate infection (Brodhagen et al. 2008, Patkar et al. 2015). In symbiotic cnidarians (Glossary: Cnidaria), oxylipins of algal endosymbionts (Symbiodiniaceae) are presumed to suppress host oxylipin expression to facilitate symbiont persistence, thereby helping maintain the cnidarian–algal symbiosis (Matthews et al. 2017, Lawson et al. 2019). So far, these studies have focused on the cnidarian–dinoflagellate relationship, but the putative role of fungi and bacteria in coral holobiont oxylipin signaling remains unexplored. Similarly, the production of volatile oxylipins in coral reef organisms and their ecological roles remain unknown but warrant further investigation.

### Volatile organic compounds

Volatile Organic Compounds (VOCs; Glossary) are small compounds that diffuse easily through water and gas and play a critical role in biosphere–atmosphere interactions, in plant signaling, and as infochemicals in multitrophic interactions (Yuan et al. 2009, Kegge and Pierik 2010). The importance of fungal VOCs in microbe–microbe and host–microbe interactions has long been overlooked, but recent studies suggest they have a significant role in long-distance signaling in bacterial–fungi interactions (Effmert et al. 2012, Schmidt et al. 2015, Jones et al. 2017). Fungal VOCs can have a wide a range of antagonistic (e.g. growth and virulence inhibition and antimicrobial properties) and mutualistic (e.g. growth promotion and secondary metabolite production) effects on other microbes (Strobel et al. 2001, Vespermann et al. 2007, Minerdi et al. 2008). However, fungi themselves are also exposed to microbial VOCs that modify their behavior. For instance, *Pseudomonas aeruginosa* produces VOCs that stimulate the growth of the opportunistic fungus *A. fumigatus*, favoring its invasion of lung tissue (Briard et al. 2016). Although the role of VOCs in marine systems remain highly unexplored, the study of “volatilomes” (Glossary), i.e. the collection of all VOCs emitted by an organism, and their putative role in organism welfare and holobiont interactions is gaining traction in coral reef studies (Swan et al. 2016, Lawson et al. 2020, Olander et al. 2021). Environmental factors such as temperature stress drive the composition and diversity of VOC emissions in coral holobionts (Lawson et al. 2021). Furthermore, the study of volatilomes from different holobiont partners suggest a complex multipartite metabolic interplay between the algal endosymbionts, their associated bacteria, and coral hosts (Lawson et al. 2021). Whilst the diversity of VOCs produced by coral-associated fungi remains yet to be charted, studies have shown that fungi can also produce important VOCs previously identified in coral holobionts, such as DMS (Bacic et al. 1998), an abundant catabolite of DMSP (Curson et al. 2011). For example, the coral pathogen *A. sydowii* is known to catabolize DMSP into DMS (Kirkwood et al. 2010). These examples highlight the importance of uncovering the diversity and putative functional roles of coral-associated fungal VOCs, which could help understand the functioning of the coral holobiont in unperturbed and stressful environments.

### Cooperative metabolite synthesis

As the different partners in a symbiosis often work collaboratively, the synthesis of some metabolites (VOCs, QS molecules or secondary metabolites) highly depends upon these complex multipartite interactions (Partida-Martinez and Hertweck 2005, Shao et al. 2020). Therefore, not only the cross-communication can affect the production of secondary metabolites by modulating gene expression or unsilencing silent BGCs (Netzker et al. 2015), but distinct organisms can also provide different substrates, enzymes and pathways that lead to cooperative metabolite biosynthesis (Cavaliere et al. 2017). Although this topic has recently been explored in microbial coculturing approaches under what is known as the OSMAC (One Strain MAny Compounds; Glossary) approach, cooperative biosynthesis *in hospite* still remains a black box (Cavaliere et al. 2017). Therefore, research efforts aiming to understand the functional role of fungi in coral holobionts should focus on understanding the contribution of the different symbiotic or commensal organisms to the “holometabolome,” i.e. the net metabolome of the holobiont, instead of individual holobiont members in isolation.

### Fungal mutualism in the ocean

#### Evidence from the fossil record to natural and experimental cocultures

Little is known about mutualistic and commensal fungal relationships in the ocean, but new symbiotic relationships are continuously being discovered (Zhang et al. 2021b, Schvarcz et al. 2022). This suggests that marine mutualistic fungi may not be absent but may have been simply overlooked until now. Fossil and extant records indicate that putative chemoautotrophic mutualistic fungi–bacterial consortia may have existed over geological timescales in the oceanic crust (Ivarsson 2012, Bengtson et al. 2014). Most notably, a few exciting examples are showcasing the high potential of fungi to form (mutualistic) symbioses with other organisms in the marine realm. This includes the recently described association of a marine sediment-dwelling fungus with its bacterial endosymbiont, the latter of which modulates the antimicrobial (polyketide) biosynthetic activity of its fungal host (Shao et al. 2020). This behavior is thought to convey a protective mechanism against other microbial competitors, which may also contribute to microbiome structuring in the sediment. A second example has described the experimental, forced coculture of the marine alga *Nannochloropsis oceanica* with the soil fungus *Mortierella elongata*. This coculturing effort not only demonstrated changes in the productivity and growth (Du et al. 2019), but also the induction of reciprocal C and N translocation between the two organisms as well as the eventual incorporation of viable algal cells within the fungal mycelium. Notably, this exciting artificially induced endosymbiosis remained stable over months of cocultivation, demonstrating a latent capacity for fungal–algal mutualism in the marine realm and providing a unique opportunity for the study of evolution of endosymbioses and fungal adaptations to novel marine hosts and environments. Such unique observations imply there is a high likelihood of coral reefs, widely known for their great functional, ecological, and taxonomic diversity, harboring a plethora of similar relationships between fungi and other reef biota. This example also impressively highlights the importance of experimental coculturing efforts for the discovery and study of marine microbial interactions.

#### The quest for probiotic potential of fungi on coral reefs

The poor outlook of coral reefs persisting in the Anthropocene (Hughes et al. 2017) is currently driving significant research efforts to explore the potential of “microbial” strategies to mitigate some of the detrimental effects of rapid global climate change (Peixoto et al. 2021). These efforts include the development of microbiome modification protocols and probiotic consortia (Glossary: probiotics) to physiologically “augment” coral holobionts with the goal of increasing their resilience to environmental perturbation, in particular ocean warming. One main goal is to maintain the coral–algae symbiosis, which can be rapidly destabilized by frequent heat wave events resulting in coral reef degradation across the world. While probiotics are already employed in marine food production (Parata et al. 2021), the development of such applications for coral reef conservation is much more recent (Rosado et al. 2018, Buerger et al. 2020, Doering et al. 2021, Dungan et al. 2021, Zhang et al. 2021a).

The probiotic potential of reef-associated fungi remains unexplored, but many fungal traits, in particular metabolic capabilities and bioactivities, align well with desired beneficial functions for coral probiotics as outlined by (Peixoto et al. 2021). For instance, the mitigation of cellular stress *via* antioxidant properties of probiotics is considered a potential strategy to alleviate the effects of heat stress in coral holobionts (Rosado et al. 2018, Dungan et al. 2021). Indeed, fungi are known to contribute to oxidative homeostasis (Glossary) in mycorrhizal symbioses (Nath et al. 2016, Huang et al. 2017). The putative roles of coral-associated fungal products, such as antioxidant enzymes (Gostinčar and Gunde-Cimerman 2018), photo-protective compounds (Sinha et al. 2007), or detoxifying enzymes (Massaccesi et al. 2002), hence warrant further investigation for their suitability in stress mitigation in corals. Furthermore, fungal antibiotics and QS molecules could be beneficial for their marine hosts *via* protection from pathogen entry (Ritchie 2006) and/or *via* fungal antifouling activity (Xu et al. 2015), which could help maintain the host-associated microbial community in times of stress. Finally, nutritional benefits provided by probiotics to sustain the marine holobiont during times of low environmental nutrient availability or environmental stress (Cardini et al. 2015) may be desirable. This is particularly important for corals that have undergone “bleaching,” a morbid state following mass expulsion of the coral's intracellular endosymbiotic algae (Strake et al. 1988). “Bleached” corals are vulnerable as they are starved of their major energy source, their algal symbionts’ photosynthate, while simultaneously being weakened by environmental stress (Rädecker et al. 2021). Here, different N cycling pathways have been implicated in the maintenance or destabilization of the coral–algae symbiosis depending on prevailing environmental conditions (Rädecker et al. 2015, Pogoreutz et al. 2017). While potential beneficial traits of endolithic fungi are yet to be characterized, pathways involved in the cycling of N (Wegley et al. 2007, Amend et al. 2012), a major element limiting productivity on oligotrophic coral reefs, could potentially help maintain nutritional homeostasis under environmental stress (Rädecker et al. 2015). In these (and potentially other) contexts, fungi could potentially play crucial roles in the health and microbiome structuring of reef-dwelling holobionts, such as corals. These potential functions warrant the exploration of coral- and reef-associated fungi in the quest for coral probiotics, which until now has focused on the algal endosymbionts (Buerger et al. 2020) and bacteria associated with corals (Rosado et al. 2018). We hence propose to include reef-associated fungi into future efforts to elucidate the untapped probiotic microbial potential, which may help deliver novel solutions for coral reef management and restoration.

### Fungal pathogens, opportunists, and parasites in the coral reef environment

#### Sea fan aspergillosis

Fungal pathogens in humans and commercially important crops have been, and still are receiving significant attention (Feurtey and Stukenbrock 2018). On coral reefs, different disease-like phenotypes have been linked to fungi (Ravindran et al. 2001, Yarden et al. 2007, Sweet et al. 2013, Soler-Hurtado et al. 2016). The probably best studied among them is a putative epizootic causing “sea fan” aspergillosis (Glossary) in octocorals (Alker et al. 2001; from here on referred to as “aspergillosis”). The first aspergillosis outbreak followed by mass mortality of *Gorgonia flabellum* and *G*. *ventalina* in the Caribbean was described in the 1980s (summarized in Smith and Weil 2004). Similar outbreaks affecting other octocorals in the tropical Atlantic and the Tropical Eastern Pacific were observed in the 1990s, and the 2000s, respectively (Smith and Weil 2004, Barrero-Canosa et al. 2013).

Symptoms of aspergillosis include tissue lesioning and recession, followed by discoloration (“purpling”) of affected tissues and gall formation. Affected tissue samples contained high loads of septate fungal hyphae, and culturing efforts identified the putative agent of the disease, a soil-dwelling saprobic fungus affiliated to *A. sydowii* and a “pollutogen” from terrestrial run-off onto coral reefs (Smith et al. 1996). Transfection experiments from infected onto healthy sea fans initially suggested aspergillosis is transmissible (Smith et al. 1996). However, recent evidence suggests *A*. *sydowii* may not be the cause of aspergillosis (Toledo-Hernández et al. 2008, 2013), but rather an opportunist invading the tissues due to declining host health (Rypien et al. 2008, Toledo-Hernández et al. 2008). Outbreaks were often linked with increases in ambient temperature (Kim and Harvell 2004), which also promotes the growth of *A. sydowii* isolates *in vitro* (Alker et al. 2001). Similarly, elevated temperatures *in situ* likely weaken coral immune defenses, permitting the proliferation of fungal opportunists (Ward et al. 2007).

The mechanisms of virulence associated with aspergillosis remain poorly understood. Phenotypic characterization of *A. sydowii* demonstrated a correlation of the secondary metabolites sydowinol, sydowinin A and B, and sydowic acid with strain pathogenicity (Smith and Weil 2004). These metabolites adversely affected the photochemical efficiency of coral-associated algal endosymbiont cultures (Symbiodiniaceae) with different symbiont types being differentially susceptible (Hayashi et al. 2016). It remains unknown whether these secondary metabolites also affect algal symbiont physiology *in hospite*, and whether this mechanism is ultimately linked to aspergillosis. Finally, pathogenic coral-associated *A*. *sydowii* strains harbor DMSP lyase *dddP*, which catalyzes the catabolism of DMSP (Kirkwood et al. 2010), a compatible solute produced in high abundances by Symbiodiniaceae, the coral (Raina et al. 2010b) and associated bacteria (Lawson et al. 2018). Coral tissues typically contain high concentrations of this compound (Raina et al. 2010b) and could, therefore, provide abundant substrate for DMSP catabolizing fungi such as *A*. *sydowii* (Kirkwood et al. 2010). It remains unclear whether the ability to catabolize DMSP confers any selective advantages to *A*. *sydowii*, such as in its ability to colonize host corals, whether it affects its pathogenicity, detoxification, or chemical signaling (Kirkwood et al. 2010), or whether the fungus merely utilizes DMSP as an environmental cue or C source. Overall, these studies suggest that fungal invaders leverage on chemical cues and crosstalk to interact with other members of the coral holobiont. Further studies will be required for a better understanding of the environmental and biotic drivers and mechanisms of fungal pathogenicity and opportunistic infection on coral reefs.

#### Enigmatic endolithic fungi of coral reefs

Coral endolithic fungal communities associated with the calcareous skeleton have been studied for decades, yet their functions remain to be fully characterized (Pernice et al. 2019, Ricci et al. 2019). Coral endolithic fungi were proposed to contribute to nutrient cycling *via* remineralization of organic matter, such as dead cells (Risk and Muller 1983, Priess et al. 2000). Endolithic fungi are largely viewed as bioeroders, parasites, or opportunistic pathogens (Yarden et al. 2007, Gleason et al. 2017) as they seemingly “attack” the filamentous, skeleton-dwelling algae *Ostreobium*, but also attempt to penetrate the live tissue layer of corals (Bentis et al. 2000). The coral host thwarts these fungal attacks by continuously accreting layers of “repair aragonite,” forming characteristic perl- or cone-like structures around the ever-probing hyphae (Bentis et al. 2000, Fig. 3). Isolates of the coral associated basidiomycete *Cryptococcus*, a genus implicated in human cryptococcosis, were shown to selectively prolong short-term survival of skeletogenic coral cell types in coculture, which was interpreted as stimulation of coral defense reactions by the presence of the fungus (Domart-Coulon et al. 2004). Interestingly, the tissues of fire corals (*Millepora complanata*; Hydrozoa) were laden with fungal hyphae following a marine heat wave resulting in “coral bleaching.” Opportunistic coral-associated saprobic fungi may be able to overcome weakened immune defenses of their vulnerable host, analogous to human secondary fungal infection in the aftermath of viral disease (Baddley et al. 2021; Fig. 3).

While metabolic interactions of endolithic fungi with other members of the coral holobiont (coral host cells, Symbiodiniaceae, or associated prokaryotes) have yet to be characterized, it may be possible they divert photosynthate from coral-associated algae (*Ostreobium*, Symbiodiniaceae), as observed in phytoplankton–chytrid pathosystems (Kagami et al. 2007b, Klawonn et al. 2021, Fig. 3). It remains to be determined whether this proposed interaction indeed occurs, and whether it is ecologically relevant in healthy corals under unperturbed conditions. During times of prolonged environmental stress however, when organic C translocation from endolithic algal communities to the coral host may become physiologically relevant (Fine and Loya 2002), depletion of such alternative C supplies by parasitic fungi could further exacerbate the health of the impaired coral host (Fig. 3). In addition, a mechanistic understanding of the potential for virulence of skeleton- or tissue-dwelling fungi under environmental perturbation is required. Increased seawater surface temperatures have been linked to the reemergence of sea fan aspergillosis (Kim and Harvell 2004), and heat or excess nutrient stress are known to induce virulence in coral bacterial pathogens (*Vibrio shilonii* and *V*. *corallilyticus*; Rosenberg and Falkovitz 2004, Kimes et al. 2012) and reef bacterioplankton (Cárdenas et al. 2018), respectively. Combined molecular, metabolomic and (cryogenic) imaging applications *in hospite* may help shed light on fungal disease and opportunism in the tissues of coral holobionts.

#### Parasitic interactions in the water column and implications for reef benthic–pelagic coupling

Reef-building corals, the main ecosystem engineers of tropical coral reefs, are mixotrophic photosymbiotic holobionts. Corals ingest pelagic organisms ranging from mesozooplankton to phyto- and bacterioplankton, which can contribute significantly to the corals’ C and N budgets and maintain coral health and resilience under environmental stress (Grottoli et al. 2006). Coral reef and pelagic food webs are thereby inevitably and intimately linked *via* benthic–pelagic coupling. This implies that changes in pelagic food web dynamics may have cascading effects on coral reef food webs and nutrient cycling, and *vice versa*.

One of the best studied examples of aquatic fungal interactions are parasitic associations of chytrids with phytoplankton (Kagami et al. 2007a, Klawonn et al. 2021). Despite the often-high proportion of infected host cells during chytrid outbreaks in some lakes (up to 90%; Kagami et al. 2006, Rasconi et al. 2012, Gerphagnon et al. 2017), fungal parasitism has been rarely considered in food web or nutrient cycling studies in other aquatic systems. However, fungal parasitism may be an integral part of aquatic food webs, as it affects fluxes of energy, nutrients, and elements (Kagami et al. 2014) and may help maintain the overall health of phytoplankton populations by selectively removing moribund and senescent cells (Laundon et al. 2021). Furthermore, fungal parasitism establishes novel trophic links by taking up organic matter from large “inedible” phytoplankton and by subsequently being consumed by zooplankton, which increases the efficiency of trophic transfer by drawing energy and nutrients up to higher trophic levels (Kagami et al. 2007b, Agha et al. 2016, Sánchez Barranco et al. 2020). Klawonn et al. (2021) demonstrated the significance of this “fungal shunt” (originally described as “mycoloop;” Kagami et al. 2006) in a model freshwater diatom–chytrid pathosystem, where fungal infection affected holobiont organic C partitioning. Diatom-associated chytrid sporangia and free-swimming zoospores met their metabolic requirements by diverting C and N from their diatom host, while organic C retained in the host cell and transfer efficiencies of C and N to associated and free-living bacteria decreased significantly. Assuming an infection prevalence of 54% in a lake phytoplankton population, up to 20% of total diatom-derived photosynthetic C would be diverted to chytrids, bypassing the microbial loop (Klawonn et al. 2021). Parasitic chytrid outbreaks can thus shape microbially mediated C and N flows at the base of aquatic food webs and accelerate biogeochemical cycles. While currently little information is available on the prevalence, severity, and ecological importance of phytoplankton–chytrid infections in the marine realm, let alone the coral reef water column, potential fungal parasite-driven changes in food web dynamics could affect the energetics of hetero- and mixotrophic coral reef filter feeders *via* benthic–pelagic coupling (Fig. 4). Specifically, parasitic chytrids bypass the microbial loop and transfer energy from phytoplankton to grazing zooplankton (Kagami et al. 2007b, Klawonn et al. 2021). Considering that tropical coral reefs typically thrive in oligotrophic waters, even minor increases in reef-associated zooplankton biomass and/or nutrient content could result in significant ecological feedback, with (potentially beneficial) nutritional effects for coral reef benthic filter feeders (Fig. 4). While speculative at this point, controlled *in vitro* and mesocosm studies combined with metabarcoding approaches characterizing pelagic fungal communities on coral reefs may help elucidate the ecological significance of (pelagic) parasitic fungi in coral reef food webs.

#### Challenges, open questions, and future directions

##### Synthesis

Fungi have conquered terrestrial, freshwater, and marine environments owing to unique sets of functional traits thought to facilitate fungal spread, diversification, and ecological adaptation. We here provided a conceptual perspective on the putative roles of understudied fungi on coral reefs by integrating our knowledge of these traits from other ecosystems and hosts with the current knowledge of fungal interactions on coral reefs, a brief summary of which is provided below.

1. In the open oligotrophic ocean, fungal biomass is low compared to phytoplankton and bacteria, and likely particle associated. This may also apply to oligotrophic tropical coral reef waters, where pelagic parasitic and saprobic fungi may affect the reef-food web *via* benthic–pelagic coupling. While fungal contributions to reef biogeochemical cycling may be of smaller magnitude compared to terrestrial environments, it could be of ecological relevance at different scales of biological organization on oligotrophic tropical coral reefs.
2. On the reef, fungi may be ecologically relevant in specific scenarios, including in reef substrata, in the reef framework, and in complex holobionts. Fungi engaging in microbe–microbe or host–microbe associations may be a driver of nutrient cycling and microbial community structuring *via* chemical mediation.
3. Fewer examples of fungal interactions are known from marine compared to terrestrial environments, most of which are of pathogenic or opportunistic nature. On coral reefs, this includes disease-like phenotypes linked to *Aspergillus* spp. as well the seemingly opportunistic behavior of coral skeleton-associated fungi. Parasitic associations with coral reef phytoplankton affecting the pelagic food web and benthic–pelagic coupling may occur but remain yet to be confirmed.
4. Based on our literature examination of fungal traits from various ecosystems and marine coculturing studies, we propose that there is appreciable potential for mutualistic fungal interactions on coral reefs (and potentially other marine environments) yet to be discovered. The lack of examples of such mutualistic associations of marine fungi with other biota may be due to a bias toward the study of fungal pathogens and opportunists, a trend noticeable also in the study of terrestrial crop and human pathogen emergence.

Taken together, we anticipate that functions of reef-associated fungi are likely diverse, spanning a spectrum of interkingdom interactions that may mediate processes at different levels of biological organization, from cell–cell interactions to ecosystem-scale effects. These may be facilitated *via* fungal chemical communication and defense, microbiome structuring as well as biogeochemical cycling, thereby extending beyond their previously reported roles as pathogens and opportunists. In the following, we briefly summarize major challenges and open questions in the study of reef-associated fungi and propose a multidisciplinary toolbox to help address these questions.

##### Open key questions and future directions

Diverse knowledge gaps remain regarding the ecology of marine fungi in coral reef environments. Broadly, outstanding questions include: how does the taxonomic and functional diversity of fungal communities in corals and on coral reefs distribute across space and time; which ecological guilds and types of (symbiotic) interactions do occur in coral holobionts and on the reef; what are the mechanistic underpinnings of microbe–microbe and host–microbe interactions that fungi engage with in coral holobionts and on coral reefs; which (a)biotic drivers govern, maintain, and alter fungal communities and interactions; *vice versa*, how do fungal interactions on coral reefs shape their (a)biotic environment from the cellular to the ecosystem level?

**Figure 5.**
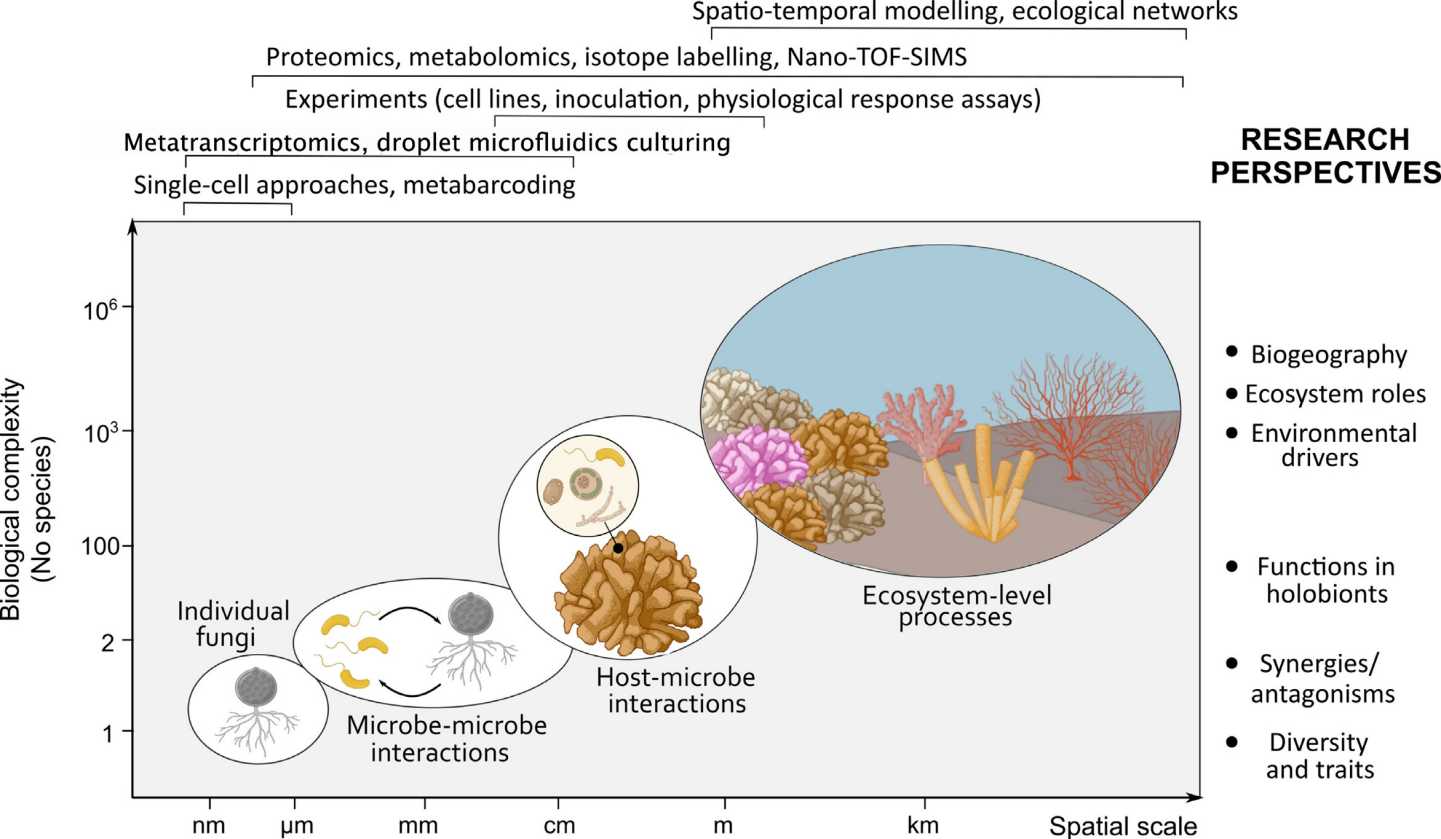
Schematic summary of key research questions and topics to improve our understanding of fungal communities and roles in coral reefs ecosystems ranked by spatial scales and biological complexity.

These questions could be addressed by employing a multipronged approach which targets different levels of biological organization for an integrated view of fungal functions in complex holobionts and the ecosystem (Fig. 5):

1. Resolving the fundamental technical challenges and streamlining of fungal community assessment workflows will aid in addressing the key question of fungal diversity, distribution, and dynamics on coral reefs. The choice of molecular markers and DNA extraction tools for fungal community characterization is inherently biased (Frau et al. 2019), highlighting the importance of workflow optimization and standardization. Developments of metabarcoding markers for marine fungi should not only aim to increase marker specificity to reduce cross-amplification (Scholz et al. 2016), but could employ a greater diversity and/or combination of markers, for instance the internal transcribed spacer (ITS; ITS2) rDNAs in conjunction with the small and large ribosomal subunits (SSU and LSU rRNA, respectively) and/or different protein-encoding regions (Tekpinar and Kalmer 2019). Such efforts could further benefit from or be complemented by the application of long-read and hybrid sequencing applications for marker genes and/or entire metagenomes (Lücking et al. 2020, Furneaux et al. 2021). Further, novel analytical frameworks to resolve genetic delineation in complex (marine) fungal communities may help solve shortcomings around the high intragenomic variance of some target regions commonly used for fungal metabarcoding, such as the ITS rDNA (Lindner and Banik 2011). A prominent example for such a framework is *SymPortal*, which resolves “defining” intragenomic variants for ITS2-type profiles of coral-associated endosymbiotic algal communities (Hume et al. 2019). Finally, the development of specific markers for fungal functional genes encoding for metabolic pathways perceived to be potentially relevant for coral and reef health could further be explored, including but not limited to selected CAZymes, DMSP lyases or genes associated with major fungal N cycling pathways. Such developments in conjunction with increased sequencing efforts and availability of genomic information may help improve community characterization at higher taxonomic levels, and ultimately allow for a more accurate assignment of functional groups (Bahram and Netherway 2022). At the same time, current efforts toward database optimization and expansion are critical for the meaningful interpretation of phylogenetic and diversity data (e.g. Martorelli et al. 2020), and will ultimately aid the prioritization to include marine fungal diversity in conservation efforts (Vatova et al. 2022).
2. Functional work on coral reef-associated fungi (and prokaryotes) is challenged by e.g. the pervasive amount of host-derived nucleic acids, which constitute a major hurdle for culture-independent sequencing approaches, as well as well-known limits to microbial cultivability (Alain and Querellou 2009, Robbins et al. 2019, Pogoreutz et al. 2022). Here, a range of technologies could be accessed to address these challenges. Laser-capture microdissection approaches could be employed to target selected fungal cells in different holobiont compartments, i.e. (coral) tissues or microenvironments such as the ectodermis, gastrodermis, mesoglea, gastric cavity, skeleton, or mucus. Such obtained samples could be used for low-input omics techniques such as single-cell genomics, transcriptomics, proteomics, or metabolomics to elucidate fungal activities and putative interactions in the intact symbiosis (Hughes et al. 2022). Further, customized microfluidics platforms (Glossary) could aim to accommodate a range of eukaryotic cell shapes (including filamentous and branched structures; Millet et al. 2019) and sizes, potentially in combination with high-throughput microbial culturing approaches to increase isolation and culturing success of slow-growing or viable, but not (currently) culturable reef-associated fungi. Such platforms may permit the application of novel coculturing or microcosm approaches mimicking small scale environments, such as the coral host environment or the phycosphere of algal symbionts, as recently established for phytoplankton-associated bacterial communities (Raina et al. 2022). Ultimately, the proposed approach may lead to the discovery of new marine symbioses, will increase the availability of genomic and functional data, may aid (co-)cultivation efforts (fungal–bacterial, fungal–fungal, fungal–microalgal, and host–fungal) for experimental interrogation (Millet et al. 2019), as detailed below, and may ultimately lead to the discovery of new marine symbioses.
3. The nature and specific mechanisms underpinning microbe–microbe interactions (fungal–bacterial and fungal–algal) could be addressed in a combination of different culture-dependent and -independent applications. High-throughput OSMAC applications on microbial cocultures (Cavaliere et al. 2017) coupled with metatranscriptomics, -proteomics, or metabolomics would not only permit the comparison of metabolic profiles, but enable an integrated assessment of chemical crosstalk in synthetic microbe–microbe associations in a range of different environmental conditions and substrates (*sensu* Presley et al. 2020). Further, anabolic turnover (Glossary: anabolism) and exchange of metabolites in these microbe–microbe associations could be mapped and quantified at (sub)cellular resolution by combining cocultures grown on isotopically labeled substrates with stable isotope probing (SIP; Glossary) and Nanoscale or Time of Flight Secondary Ion Mass Spectrometry (Nano- and ToF-SIMS; Glossary; Raina et al. 2017). Specifically, cultures of algae, bacteria, and fungi originally isolated from coral and other reef holobionts could be labeled separately with distinct isotopes (e.g. ^13^C, ^15^ N, and ^34^S) prior to unlabeled coculture, and subsequently preserved and prepared for correlative electron microscopy and SIMS analysis to visualize (sub)cellular assimilation and distribution of isotopic labels and metabolic interactions between cells. Such approaches, especially in combination with correlative fluorescence *in situ* hybridization (FISH; Glossary) and omics applications as outlined above, would allow for the assessment of interactions in natural microbe–microbe assemblages or in clearly defined, synthetic communities of reduced complexity by targeting specific microbial functional groups or combinations of taxa. Notably, cultures of major coral-associated algal symbionts are available at dedicated microbial culture collections and can be readily maintained in stable cultures. Similarly, the availability of well-characterized coral bacterial isolates is steadily increasing, facilitating functional studies of microbe–microbe interactions *in vitro* (Sweet et al. 2021, Pogoreutz et al. 2022).
4. Another interesting venue is the study of coral reef host–fungal interactions, which can be approached at either the cellular or the organismal level. In recent years, cell lines of corals, anemones and sponges have become available (Domart-Coulon et al. 2001, Ventura et al. 2018), which can be leveraged for functional laboratory model-system approaches in nonmodel reef organisms at the cellular level. Such an approach employing a methodological toolbox as described in (3) would have multiple benefits: the characterization and visualization of real-time host–microbe interactions in simplified, defined holobionts (one host, one or multiple selected microbes) and the study of metabolic interactions without the confounding effects of host–host cell interactions. The use of stable isotope labeling approaches to target specific metabolisms and trace the fate of specific molecules or substrates (e.g. fungal assimilation, fungal–host translocation, or *vice versa*), as detailed for the study of complex multipartite host–microbe interactions (Lê Van et al. 2016, Rädecker et al. 2021) may here be particularly suitable. At the organismal level, understanding the roles of different fungi in the coral holobiont (e.g. mutualists, opportunists, and pathogens) will be central for our understanding of coral holobiont health and resilience to different environmental stresses. For example, mutualistic fungal strains could potentially be used in novel probiotic and reef restoration applications, while an understanding of identity, drivers and mechanisms of fungal pathogenicity may inform the diagnosis, mitigation, or possibly even prevention of coral disease outbreaks *via* meaningful management tools. Here, host inoculation experiments with potential mutualistic and/or pathogenic strains could be used for functional interrogation. Specifically, a combination of in-depth physiological phenotyping assays (e.g. photophysiological parameters, algal symbiont densities *in hospite*, oxygen fluxes, nutrient uptake and release dynamics, oxidative stress, and so on; Rosado et al. 2018, Doering et al. 2021, Rädecker et al. 2021) and (meta)transcriptomic, (meta)proteomic, or (holo)metabolomic assessments (*sensu* Mohamed et al. 2018, Santoro et al. 2021) of the host or holobiont could be employed. Another perspective which warrants exploration is the notion that the microbial community may be able to modulate coral host phenotypic responses *via* epigenetic modification (Barno et al. 2021). Fungi exhibit epigenetic interactions with plant hosts (de Palma et al. 2019). It will, hence be of interest to characterize whether coral-associated fungi are capable of causing epigenetic changes in immune or environmental response genes in their host, whether such epigenetic changes result in distinct host phenotypes, and whether they may affect host resilience to environmental stress. Together, the proposed approaches may help elucidate not only the nature of the association of diverse fungi with their hosts, but also of the fungal potential to mitigate holobiont stress in global change scenarios.
5. Finally, assessing the roles that fungi play at the community and ecosystem-levels and their spatio-temporal dynamics will be critical to advance the knowledge of fungal functions in coral reef ecosystems. Understanding and predicting how changes in coral reef fungal communities might translate into community-scale cascade effects and shifts should become a major goal. These include but are not limited to the (a) determination of (a)biotic drivers of fungal distribution and abundance in space and time, (b) quantification of fungi-specific activities at the community level such as their involvement in nutrient and energy transfer within and between the benthic and the pelagic reef environment, and (c) modeling of community-level budgets (e.g. C or N budgets) as well as ecological networks under consideration of fungal activity. In terrestrial environments, climate has been often reported as one of the strongest drivers affecting fungal community composition (Cavicchioli et al. 2019, Egidi et al. 2019), therefore, a considerable challenge will be to understand the dynamics and biogeography of reef fungal communities. Disentangling how anthropogenic impacts and climate change affect both the structure and functioning of reef fungal communities will be paramount and will inevitably require modeling the effects of changing environmental conditions on biotic interactions (e.g. changes in use of resources, competition, or pathogenicity). To tackle such a challenging endeavor, it will be necessary to identify and quantify rates of specific fungal metabolisms of interest across different benthic and pelagic coral reef microhabitats. This can for instance be achieved by systematic and high-resolution sampling campaigns at smaller spatial and temporal scales, which can be generated through *in vitro* (on cultures), *ex situ* (in holobionts, such as individual corals or polyps), or *in situ* (on structurally complex benthic communities) experiments, as recently established for rate measurements of selected prokaryotic or holobiont-level metabolisms, such as dinitrogen fixation or oxygen fluxes (Cardini et al. 2016, Roth et al. 2019). *In situ* set-ups could be equipped with multiple sampling ports for the controlled and reproducible sampling of different biological and chemical variables (Roth et al. 2019), and employed in either natural (e.g. comparison of metabolic rates between coral reef sites with different degrees of anthropogenic disturbance or natural variation, such as thermal fluctuations) or manipulative experiments (e.g. *in situ* simulation of major environmental stressors, such as global warming, ocean acidification, or eutrophication). Such approaches may hold the promise to address the work outlined above in a) to c), to help elucidate fungal contributions to reef-scale biogeochemical cycling.

### Glossary

#### Anabolism vs. catabolism

The former is the synthesis of complex molecules (i.e. catabolites) from simpler ones, which requires energy, whereas the latter is the breakdown of complex molecules into simpler ones (i.e. catabolites) releasing energy.

#### Antagonistic vs. synergistic interactions

The former describes a cumulative effect, i.e. less than additive, i.e. less than the sum of effects (for instance, by stressors or organisms acting in isolations). The latter defines a cumulative effect greater than the additive sum of effects.

#### Aspergillosis

A disease caused by fungi of the genus *Aspergillus*. These fungi can infect a wide range of hosts, ranging from coral to humans.

#### Benthic–pelagic coupling

Processes that connect the sea floor (i.e. the benthic zone) and the water column (i.e. the pelagic zone) through the exchange of energy, mass, or nutrients. It plays a prominent role in nutrient cycling and energy transfer in aquatic food webs, and thereby ecosystem processes. BGC: tightly linked sets of (mostly) nonhomologous genes participating in a common, discrete metabolic pathway. The genes are arranged in physical proximity to each other on the genome, and their expression is often coregulated. Common in bacterial and fungal genomes and most widely known for the production of secondary metabolites.

#### CAZymes

Carbohydrate-active enzymes which build and break down complex carbohydrates and glycoconjugates for a large body of biological roles (collectively studied under the term of Glycobiology).

#### Chemotaxis (or chemotactic behavior)

Movement of a cell or organism in response to an environmental diffusible chemical substance.

#### Chytridiomycota (chytrids)

Unicellular or mycelic, aerobic zoosporic fungi that operate as saprotrophs and pathogens in freshwater, brackish, and marine habitats.

#### Cnidaria

A phylum within the animal kingdom which includes jellyfish, anemones, and reef-building corals. Cnidaria are simple, multicellular organisms characterized by two main cell layers (ectodermis and endodermis) and an apparatus consisting of stinging cells (cnidocytes) for prey capture and defense.

#### Dimorphic switching

The ability of several fungi to switch between a multicellular hyphal and unicellular yeast morphology and growth form. The mechanism underlying this biological reorganization process depends on external (environmental/chemical) triggers.

#### Dimethylsulfonioproprionate (DMSP)

An organosulfur compound produced in vast quantities by phytoplankton and seaweeds, and known to have osmoprotectant and antioxidant function. DMSP is an important carbon source for many marine bacteria, which can break it down *via* the DMSP demethylation or DMSP cleavage pathways.

#### Ecological guilds

Any group of species that exploit the same resources, or that exploit different resources in related ways. Among fungi, common guilds are decomposers (saprobic), pathogens, endophytes, and mycorrhiza.

#### Endolithic (microbial) communities

A group of organisms including cyanobacteria, fungi, algae, and bacteria that dwell in the pore space of rocks and similar substrates, such as coral skeletons.

#### FISH

A molecular technique that uses fluorescent probes that bind to only particular parts of a nucleic acid (DNA or RNA) sequence with a high degree of sequence complementarity. Widely used in the field of microbial ecology to identify taxa and to visualize the distribution and proportion of specific taxa within environmental samples.

#### MALDI-TOF

Matrix Assisted Laser Desorption Ionization coupled to Time-Of-Flight mass spectrometry.

#### Microbialization

The observed shift in ecosystem trophic structure toward higher microbial biomass and energy use. On coral reefs, causes of microbialization include overfishing and eutrophication.

#### Microfluidics

Refers to the behavior, precise control, and manipulation of fluids geometrically constrained to a small scale. In (micro)biology, it offers a powerful approach to control the complete cellular environment, thereby enabling the study of microbial community microscale organization, cellular behavior, adaptation, or gene expression.

#### Mycorrhizal fungi

Mycorrhizae are soil-borne fungi closely associated with the roots of terrestrial plants. Arbuscular mycorrhiza colonize the intercellular spaces of plant roots (in contrast to ectomycorrhizal fungi). Arbuscular mycorrhizae are considered vital endosymbionts of plant holobionts, as they enhance productivity.

#### Next generation sequencing

Sequencing is the process of determining the order of nucleotides in entire genomes or targeted regions of DNA or RNA. Next-generation sequencing (NGS) is a technology that offers ultrahigh throughput, scalability, and speed, and includes applications such as metabarcoding (deep sequencing of target regions) for whole community studies, whole genome sequencing, RNA sequencing, or the assessment of genome-wide DNA methylation and DNA–protein interactions.

#### Oxidative stress and oxidative stress responses

The former is the imbalance between the systemic manifestation of reactive oxygen species (ROS) and a biological system's ability to readily detoxify the reactive intermediates or to repair the damage resulting in cellular components, including proteins, lipids, and the DNA. Oxidative stress responses encompass the production of antioxidant enzymes (including but not limited to superoxide dismutase, catalases, and peroxiredoxins), which aim to strike a balance between ROS production and consumption.

#### Oxylipins

Lipids, often bioactive, generated by the oxidation of polyunsaturated fatty acids (PUFAs).

#### VOCs

Compounds with a high vapor pressure and low water solubility. Although VOCs gas can be emitted from different solids and liquids, in this manuscript we only refer to VOCs of biogenic origin (i.e. produced by living organisms).

#### Volatilome

Study of all the VOCs that are produced by a biological matrix (organism and ecosystem).

#### OSMAC

One Strain MAny Compounds is an approach, which by altering cultivation parameters (e.g. medium composition, physical properties, or strain coculture), aims to activate silent BGCs and expand or modify the metabolite production fingerprints of microbial strains.

#### Probiotics

Live microorganisms with beneficial qualities for a host/recipient organism. Probiotic microorganisms help to restore recipient health by antagonistic action against pathogenic microbes, or enhance performance and growth by providing nutritional benefits to the recipient.

#### Recalcitrant vs. labile

Describes the bioavailability for or timescales of degradation of organic matter by organisms, which is reflected in the timescales by which this matter is respired. Organic matter follows a spectrum of recalcitrant (degraded slowly over years to decades, or resistant to degradation by microbes) to labile (rapidly degraded, within minutes to hours).

#### Secondary ion mass spectrometry (SIMS)

A technique used to analyze the composition of solid surfaces and thin films, which permits the spatial mapping of atoms or molecules. In correlation with electron microscopy increasingly used in biological research to create isotopic maps of histological sections, which can be used to visualize the assimilation and translocation of nutrients within complex symbiotic systems, such as corals.

#### SIP

A technique in microbial ecology for tracing uptake of nutrients by microbes. A substrate is enriched with a heavier stable isotope, i.e. consumed by the organisms to be studied. Biomarkers with the heavier isotopes incorporated into them can be separated from biomarkers containing the more naturally abundant lighter isotope by buoyant density centrifugation. As an example, ^15^N_2_ can be used to find out which microbes are active nitrogen fixers.

#### Transcription

Process of making an RNA copy (mRNA) of a gene's DNA.

## Supplementary Material

fuac028_Supplemental_File
