# Supplementary material for: A roadmap to understanding diversity and function of coral reef-associated fungi
Source: FEMS Microbiol Rev. 2022 Jun 23;46(6):fuac028. doi: 10.1093/femsre/fuac028 (PMC9629503; doi:10.1093/femsre/fuac028)
Supplement: fuac028_Supplemental_File [file fuac028_supplemental_file.docx]

# **Supplementary materials**

**Table S1. Summary of fungal taxa frequently reported from coral.** Table (A) summarizes several Ascomycota, (B) Basidiomycota, and (C) other fungal phyla or closely related subkingdoms. Diversity data on coral-associated fungi are still sparse, but taxa consistently recovered from corals across geographic locations.

| **A) Subkingdom/Phylum: Ascomycota** | | | | | |
| --- | --- | --- | --- | --- | --- |
| The phylum Ascomycota is the most frequently reported taxon found in coral samples. In many studies Ascomycota members dominated > 90% of the coral-associated fungal community (Wegley *et al.* 2007, Vega Thurber *et al.* 2009, Góes-Neto *et al.* 2020, Amend *et al.* 2012, Bonthond *et al.* 2018, Lifshitz *et al.* 2020, Chavanich *et al*. 2022) | | | | | |
| *Class* | *Order* | *Family* | *Genus / Species* | *Studies* | *comments* |
| Sordariomycetes |  |  |  | Wegley *et al*. 2007, Góes-Neto *et al*. 2020, Amend *et al.* 2012, Cárdenas et al. 2022 | Relative abundance < 70%; comprises major members of the coral mycobiome in Amend *et al.* 2012 |
|  | Lulworthiales | Lulworthiaceae | e.g. *Lindra* sp. | Góes-Neto *et al*. 2020, Amend *et al* 2012, Bonthond *et al*. 2018, Kohlmeyer *et al*. 1989, 2000 | Relative abundances > 50%; obligatory marine fungus in Kohlmeyer *et al.* 2000; they typically occur in various marine environments, coralline algae, sand, driftwood, sea foam; they are conspicuous due to abundance and prevalence in corals, e.g., in *Siderastrea siderea* across different depths; core member of coral fungal communities in Amend *et al.* 2012 and Bonthond *et al.* 2018; most abundant fungal sequence in Bonthond *et al.* 2018 |
|  | Magnaporthales | Magnaporthaceae |  | Vega Thurber *et al.* 2009 | Related taxa are known for being phytopathogens (Dean *et al.* 2005) |
|  | Ophiostomatales | Ophiostomataceae | *Hyalorhinocladiella* sp. | Bonthond *et al.* 2018 | Not overabundant but prevalent in Bonthond *et al.* 2018 |
|  | Xylariales | Hyponectriaceae | *Physalospora* spp | Bonthond *et al.* 2018 | Not overabundant but prevalent in Bonthond *et al.* 2018 |
|  |  | Sporocadaceae | *Pestalotiopsis spp.* | Paulino *et al.* 2020 | Three species were isolated from coral |
|  |  | Xylariaceae | *Xylaria* spp. | Paulino *et al*. 2020 | Isolated from coral in Brazil |
|  | Sordariales | Chaetomiaceae |  | Lifshitz *et al.* 2020 | Isolated and cultured taxon from coral samples |
|  | Microascales | Microascaceae |  | Lifshitz *et al.* 2020 | Isolated and cultured taxon from coral samples |
|  | *Incertae sedis* | Apiosporaceae |  | Lifshitz *et al.* 2020 | Isolated and cultured taxon from coral samples |
|  | Hypocreales | Nectriaceae |  | Lifshitz *et al.* 2020 | Isolated and cultured coral fungus, but also isolated from sediments/water from the sampling site! |
|  |  | Stachybotrys |  | Lifshitz *et al*. 2020 | Isolated and cultured taxon from coral samples |
|  |  | Hypocreaceae | *Trichoderma* spp. | Lifshitz *et al.* 2020, Paulino *et al.* 2020 | Isolated and cultured coral fungus (Lifshitz *et al.* 2020); four species isolated from coral in Brazil (Paulino *et al.* 2020) |
|  |  | Bionectriaceae | *Clonostachys rosea* | Paulino *et al*. 2020 | Isolated and cultured taxon from coral samples |
|  |  | Cordycipitaceae | *Cordyceps* spp*.* | Paulino *et al.* 2020 | Isolated and cultured taxon from coral samples |
| Saccharomycetes | Saccharomycetales | Saccharomycetaceae |  | Wegley *et al.* 2007, Lifshitz *et al.* 2020, Chavanich *et al*. 2022, Cárdenas et al. 2022 | A major taxon in corals (Wegley *et al.* 2007); culturable and prevalent in corals during winter (Lifshitz *et al.* 2020) |
|  |  | Saccharomycetaceae | *Candida* spp. | Rabbani *et al.* 2021, Paulino *et al.* 2020 | Abundant in few samples (Rabbani *et al.* 2021); two *Candida* species isolated from corals in Brazil (Paulino *et al.* 2020) |
|  |  | Saccharomycetaceae | *Hyphopichia* spp. | Paulino *et al.* 2020 | Isolated and cultured taxon from coral samples |
| Schizosaccharomycetes | |  |  | Wegley *et al.* 2007, Amend *et al.* 2012 |  |
| Dothideomycetes | Capnodiales | Teratosphaeriaceae | *Hortaea werneckii* spp. | Amend *et al.* 2012, Bonthond *et al.* 2018, Rabbani *et al.* 2021, Cárdenas et al. 2022 | Putative core fungal community members of corals (Bonthond *et al.* 2018, Amend *et al.* 2021); also reported in Rabbani *et al. 2*021 |
|  | Capnodiales | Davidiellaceae | *Cladosporium* spp. | Lifshitz *et al.* 2020, Rabbani *et al.* 2021, Paulino *et al.* 2020 | Isolated and cultured coral fungus (Lifshitz *et al.* 2020), can be abundant in metabarcoding data (Rabbani *et al.* 2021); several *Cladosporium* species isolated from corals in Brazil (Paulino *et al.* 2020) |
|  | Pleosporales | Sporormiaceae |  | Lifshitz *et al*. 2020 | Isolated and cultured taxon from coral samples |
|  | Capnodiales | Mycosphaerellaceae | *Mycosphaerella* spp. | Rabbani *et al*. 2021 | Abundant in metabarcoding data |
|  | Pleosporales | Pleosporaceae |  | Lifshitz *et al.* 2020 | Isolated and cultured coral fungus, also isolated from sediments/water from the sampling site |
|  |  | Pleosporaceae | *Alternaria* spp. | Rabbani *et al.* 2021 | Coral associated taxon (metabarcoding study) |
|  |  | Pleosporaceae | *Stemphylium* spp. | Rabbani *et al.* 2021 | Coral associated taxon (metabarcoding study) |
|  |  | Didymellaceae |  | Lifshitz *et al.* 2020 | Isolated and cultured taxon from coral samples |
|  |  | Didymellaceae | *Phoma* sp. | Paulino *et al.* 2020 | Isolated and cultured taxon from coral samples |
|  |  | Teichosporaceae | *Byssothecium* spp. | Rabbani *et al.* 2021 | Abundant in metabarcoding data (Rabbani *et al.* 2021) |
|  |  | Lophiostomataceae | *Lophiostoma* spp. | Rabbani *et al.* 2021 | Abundant in metabarcoding data, but only present in few samples (Rabbani *et al.* 2021) |
|  |  | Didymosphaeriaceae | *Microsphaeropsis* spp. | Paulino *et al.* 2020 | Isolated and cultured coral fungus |
|  |  | Pyrenochaetopsidaceae | *Pyrenochaetopsis* spp. | Paulino *et al.* 2020 | Isolated and cultured coral fungus |
| Eurotiomycetes | Eurotiales |  |  | Wegley *et al.* 2007, Góes-Neto *et al*. 2020, Amend *et al.* 2012, Cárdenas et al. 2022 | Members are known human pathogens; associated with 'aspergillosis' in gorgonian corals in the Caribbean (Alker, Smith and Kim 2001b), but also found in healthy corals (Soler-Hurtado *et al.* 2016) |
|  |  | Trichocomaceae | *Aspergillus* *sydowii*/spp. | Rabbani *et al.* 2021, Paulino *et al.* 2020 | Abundant in metabarcoding data (Rabbani *et al.* 2021); several *Aspergillus* species were isolated from corals (Paulino *et al.* 2020); ubiquitously found across several genera of healthy corals (Bentis, Kaufman and Golubic 2000); probably pathogenic or opportunistic |
|  |  | Trichocomaceae | e.g., *Penicillium* spp. | Rabbani *et al.* 2021, Wegley *et al.* 2007, Lifshitz *et al*. 2020, Paulino *et al.* 2020 | High abundance of Trichocomaceae found in Lifshitz *et al.* 2020); most abundant taxon isolated from *Acropora loripes*. Suggested core microbiome member, but also known to be culturable from marine sediments (Wegley *et al.* 2007, Lifshitz *et al.* 2020); six species isolated from corals (Paulino *et al.* 2020) |
|  |  | Trichocomaceae | *Talaromyces* spp. | Rabbani *et al.* 2021 |  |

| **B) Subkingdom/Phylum: Basidiomycota** | | | | | |
| --- | --- | --- | --- | --- | --- |
| Members of the Basidiomycota are commonly reported from coral-associated fungal communities (Wegley *et al*. 2007, Góes-Neto *et al*. 2020, Amend *et al*. 2012, Bonthond *et al*. 2018, Chavanich *et al*. 2022; Cárdenas et al. 2022) | | | | | |
| *Class* | *Order* | *Family* | *Genus / Species* | *Studies* | *comments* |
| Ustilaginomycetes |  |  |  | Amend *et al.* 2012 |  |
| Agaricomycetes |  |  |  | Amend *et al.* 2012, Cárdenas et al. 2022 |  |
|  | Agaricales | Physalacriaceae | *Flammulina* spp. | Rabbani *et al.* 2021 | Abundant in metabarcoding study |
|  | Agaricales | Schizophyllaceae | *Schizophyllum* spp. | Rabbani *et al.* 2021 | Abundant in metabarcoding study, but present in only few samples |
| Malasseziomycetes | Malasseziales | Malasseziaceae | *Malassezia globosa*/ spp. | Amend *et al.* 2012, 2014, Chavanich *et al*. 2022 | Majority of fungi living on/in apparently healthy colonies; significantly higher abundances amongst corals located in warmer waters (Amend *et al.* 2012) |
| Microbotryomycetes | Sporidiobolales | Sporidiobolaceae |  | Lifshitz *et al*. 2020 | Isolated and cultured coral fungus, prevalent in corals during winter season (yeast-like morphology) |

| **C) Other Subkingdoms/Phyla** | | | | | | |
| --- | --- | --- | --- | --- | --- | --- |
| *Phylum* | *Class* | *Order* | *Family* | *Genus / Species* | *Studies* | *comments* |
| Mucoromycota |  |  |  |  | Góes-Neto *et al.* 2020, Cárdenas et al. 2022 |  |
|  |  | Mucorales | Mucoraceae |  | Lifshitz *et al.* 2020, Cárdenas et al. 2022 | Fungi isolated from coral, also detected by amplicon sequencing |
| Chytridiomycota | Chytridiomycetes |  |  |  | Wegley *et al.* 2007, Vega Thurber *et al.* 2009, Góes-Neto *et al.* 2020 | Relative abundances increased with nutrient enrichment in Vega Thurber *et al.* 2009 |
| Monoblepharidomycota | Monoblepharidomycetes | Monoblepharidales | Harpochytriaceae | *Harpochytrium* spp. | Vega Thurber *et al.* 2009 | Relative abundances increased with nutrient enrichment in Vega Thurber *et al.* 2009; a zoosporic fungus that attaches to living algae (Atkinson 1909) |
| Entomophthoromycota | Entomophthoromycota |  |  |  | Bonthond *et al.* 2018 | Indicator taxon for deeper corals (17 m depth) as compared to shallow reef |
| Mesomycetozoa | Mesomycetozoa |  |  |  | Bonthond *et al.* 2018 | Fungi-like Opisthokonta (Mesomycetozoa) were present in corals |

**Table S2.** Summary of methodologies used to assess the diversity and community composition of coral-associated fungi.

| **Study** | **Microenvironment (sampling method)** | **Holobiont Condition** | **Approach** | **Sequencing method** | **Target region** | **Primer name** |
| --- | --- | --- | --- | --- | --- | --- |
| Cárdenas et al. 2022 | Skeleton (skeleton isolated by Dremel, mortar & pestle homogenization) | Healthy; Heat-stressed | cultivation-independent | Illumina HiSeq | genomic DNA  (metagenomic sequencing) | NA |
| Chavanich *et al.* 2022 | Pooled surface mucus layer, tissue, and skeleton (mortar & pestle homogenization) | Healthy; Bleached | cultivation-independent | Illumina MiSeq | 18S rRNA (SSU) | Illumina_Euk_1391F -lllumina_EukBr |
| Rabbani *et al.* 2021 | Pooled surface mucus layer, tissue (sprayed off tissue) | Healthy | cultivation-independent | Illumina MiSeq | ITS | ITS1F-ITS2 |
| Góes-Neto *et al.* 2020 | Limestone substrate and skeleton (tissues removed by chisel) | Reef substrate and healthy coral | cultivation-independent | Illumina MiSeq | 18S rRNA (SSU) | NF1/18 Sr2b |
| Lifshitz *et al.* 2020 | Pooled surface mucus layer, tissue, and skeleton (crushed with hammer) | Healthy; Lesioned | cultivation-dependent | Sanger | 18S rRNA (SSU) | KF1-KR1 |
| Paulino *et al.* 2020 | Pooled surface mucus layer, tissue, and skeleton (cut into pieces) | NA | cultivation-dependent | Sanger | ITS | ITS1-ITS4 |
| Bonthond *et al.* 2018 | Pooled surface mucus layer, tissue, loosely attached particles (high-pressure airbrushing) | Healthy | cultivation-independent | Illumina MiSeq | ITS2 | ITS3-ITS4 |
| Amend *et al.* 2012 | Pooled surface mucus layer, tissue, and skeleton (NA) | NA | cultivation-independent | Pyrosequencing 454 | D1-D2 LSU | LRORf-LR5f |
| Vega Thurber *et al.* 2009 | Pooled surface mucus layer, tissue, and skeleton (mortar & pestle homogenization) | Healthy; Exposed to 1) elevated nutrients, 2) elevated temperature, 3) decreased pH | cultivation-independent | Pyrosequencing 454 | genomic DNA  (metagenomic sequencing) | NA |
| Wegley Kelly *et al.* 2007 | Pooled surface mucus layer, tissue, and skeleton (mortar & pestle homogenization) | NA | cultivation-independent | Pyrosequencing 454 | 18S rRNA (SSU) | EukA-F-EukB-R |
| Kohlmeyer *et al.* 1989, 2000 | Limestone substrate and skeleton (tissues removed by chisel) | Reef substrate and live coral | cultivation-dependent | Sanger | SSU and LSU rDNA | NS1 and NS4 \| LR0R and LR5 |

###### **Table S3** Fungal traits proposed to facilitate the colonization of marine ecosystems and holobionts.

| **Trait** | **References** |
| --- | --- |
| **Osmotolerance** | |
| Cell wall fluidity through fatty acid modifications | Turk *et al.* 2004, Plemenitaš *et al.* 2014 |
| Cell rigidity and pressure tolerance through cell wall melanization | Kogej *et al.* 2006, 2007, Cordero *et al*. 2017 |
| Adaptation of wall composition through adjustment of the carbohydrate:amino acid:fatty acid ratio | Szaniszlo & Mitchell, 1971, Danilova *et al.* 2020 |
| Production of compatible organic solutes for osmoregulation (i.e., increased contents of saccharides and/or polyols) | Ravishankar *et al.* 2006, Gonsalves & Nazareth 2020 |
| Modifications of high-osmolarity-glycerol (HOG) signaling pathway | Turk *et al*. 2002 |
| Increased expression of salt efflux pump protein genes | Kogej *et al*. 2005 |
| High G+C content (often linked with halotolerance in prokaryotes) | Jacob 2012 |
| High levels of antioxidants | Gostinčar *et al*. 2018 |
| **Colonization of new habitats and hosts** | |
| Dimorphic switching: multicellular hyphal & unicellular yeast-like lifestyles | Kylie and Andrianopoulos 2015 |
| Motility and chemotaxis (e.g., motile asexual zoospores in Chytrids) | Scholz *et al*. 2017, Laundon et al. 2021 |
| Sensing of (physical and chemical) environmental cues | Powell, 1983 |
| Camouflage: Masking fungal signature by remodeling of cell walls and evade detection by the host’s immune system | Nagy *et al.* 2017, Klis *et al*. 2009 |
| Reciprocal genetic reprogramming *via* host-symbiont gene co-expression between host and mycorrhizal colonizer | Mateus *et al*. 2019 |
| **Nutrient acquisition & turn-over of matter** | |
| Mucilaginous sheaths, or spore walls for substrate attachment | Jones 2006 |
| High diversity of carbohydrate-active enzymes (CAZymes) facilitates flexible nutritional strategies (e.g., glycoside hydrolases) | Lange *et al.* 2019, Janusz *et al*. 2017 |
| Capability to metabolize marine recalcitrant polysaccharides from algal-derived POM, laminarin, fucoidan, porphyrin (e.g., through removal of sulfate groups in marine substrates) | Barbosa 2019, Kappelmann *et al.* 2019, Cunliffe *et al*. 2017 |
| Capability to metabolize other recalcitrant organic matter: humic acids, lignin, celluloses, tunicin, chitin | Kohlmeyer & Kohlmeyer 1989, Castaño *et al*. 2021, Tang *et al.* 2006 |
| Lectins: Carbohydrate binding proteins | Singh *et al*. 2011 |
| Extracellular polysaccharides for nutrient binding | Kimura *et al.* 1998 |
| **Secondary metabolites as means of chemical communication and interaction** | |
| Secondary metabolites with bioactivity as means for chemical communication, defense, and competition | Bahram *et al*. 2018, Keller 2019, Pierce *et al.* 2021 |
| Secondary metabolite production pathways involving large multimodular enzymes (e.g., polyketide synthases PKSs, nonribosomal peptide synthetase NRPSs, prenyltransferases and terpene cyclases) | Brakhage *et al.* 2011, Keller 2019 |
| Transcription flexibility of biosynthetic Gene Clusters (BGCs) coding for enzymes for secondary metabolite synthesis | Shostak *et al*. 2020, Keller 2019 |
| **Genomic features and adaptability** | |
| Rapid genome evolution through genome compartmentalization, extensive sequence divergence, distinct chromosome organization, | Soanes & Richards 2014, Möller & Stukenbrock 2017, Stajich 2017 |
| Rapid genome evolution through transposable elements | Hess *et al* 2014, Miyauchi *et al*. 2020 |
| Rapid genome evolution through frequent horizontal gene and viral transfer (HGT and HVT) | Bian *et al.* 2020, Wang *et al.* 2021b |
| Interspecific gene exchange *via* hybridization | Roper *et al.* 2011, Stukenbrock 2016 |
| Gene repertoire enriched in transporters (e.g., in a marine yeast) | Lépingle *et al.* 2000 |
| Higher genomic coding density (e.g., gene duplications / reduction of non-coding regions) | Dujon *et al*. 2004 |
| Environment-specific transcriptional responses: Acclimation through ‘frontloading’. | Wang *et al*. 2021a |
